# Supplementary figures and images for: Random-effects meta-analysis of effect sizes as a unified framework for gene set analysis
Source: PLoS Comput Biol. 2022 Oct 5;18(10):e1010278. doi: 10.1371/journal.pcbi.1010278 (PMC9576052; doi:10.1371/journal.pcbi.1010278)

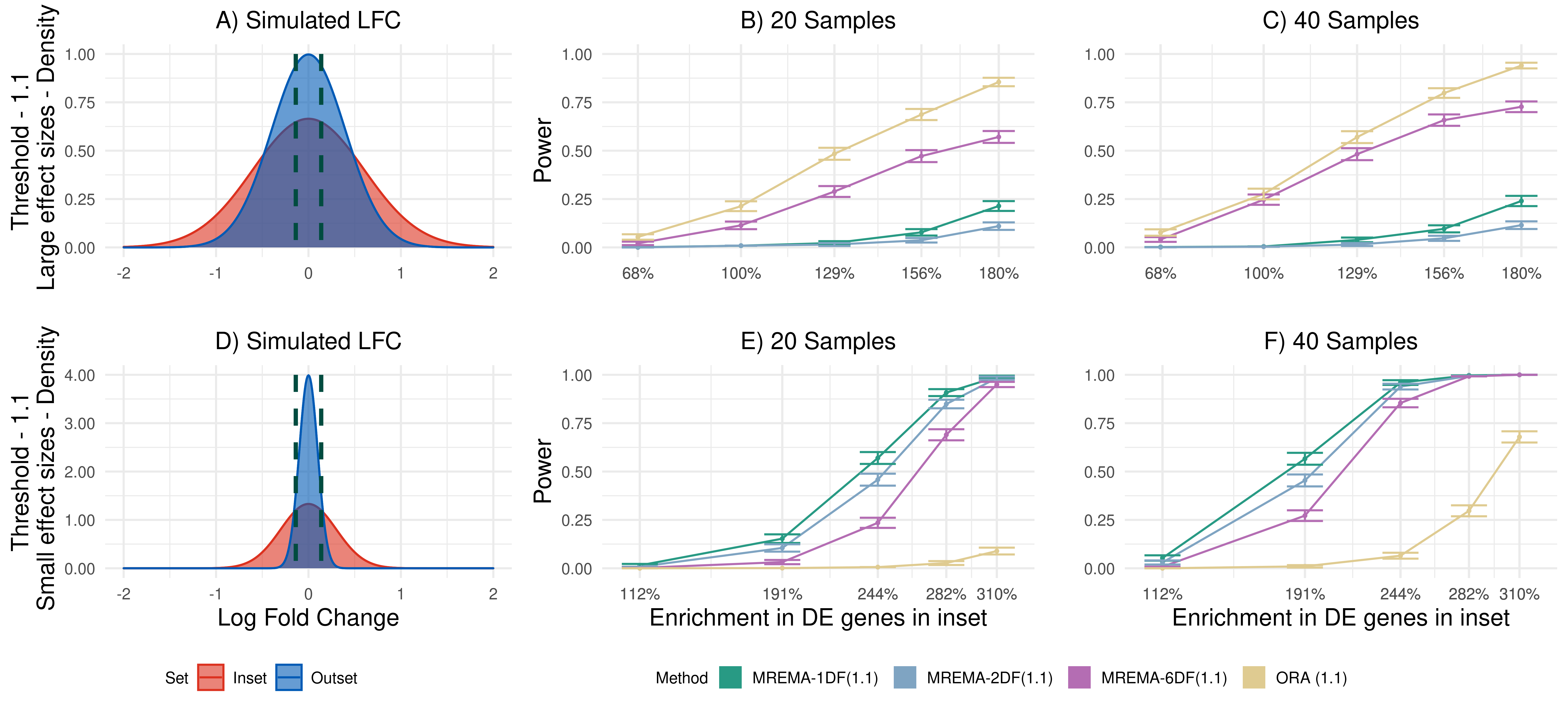

Supplement: S1 Fig — The power for the different tests is shown using a FC threshold of 1.1. The power for high LFC values illustrated in A) is shown in B) for 20 samples and C) for 40 samples. The power for low LFC values, illustrated in D) is shown in E) for 20 samples and F) for 40 samples. (TIF) [file pcbi.1010278.s001.tif]

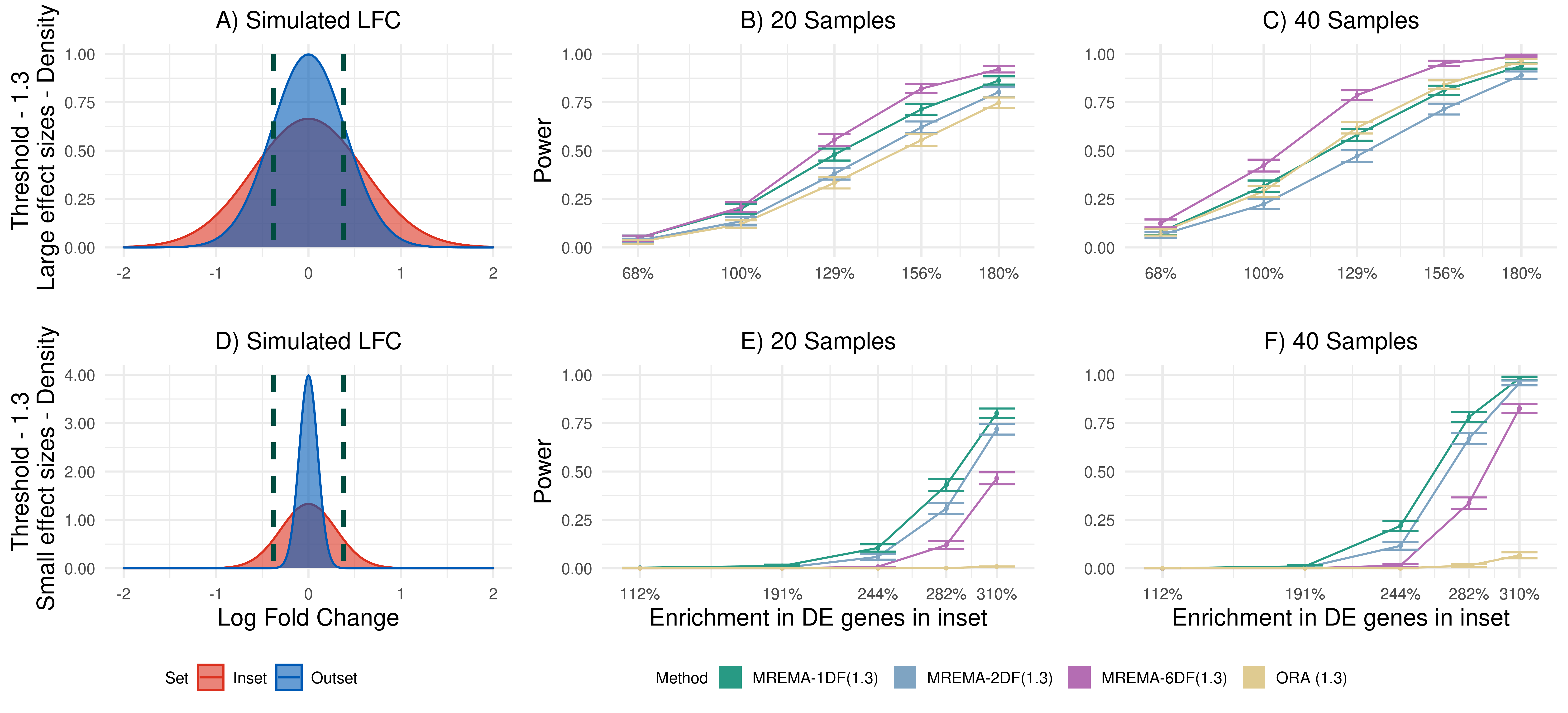

Supplement: S2 Fig — The power for the different tests is shown using a FC threshold of 1.3. The power for high LFC values illustrated in A) is shown in B) for 20 samples and C) for 40 samples. The power for low LFC values, illustrated in D) is shown in E) for 20 samples and F) for 40 samples. (TIF) [file pcbi.1010278.s002.tif]

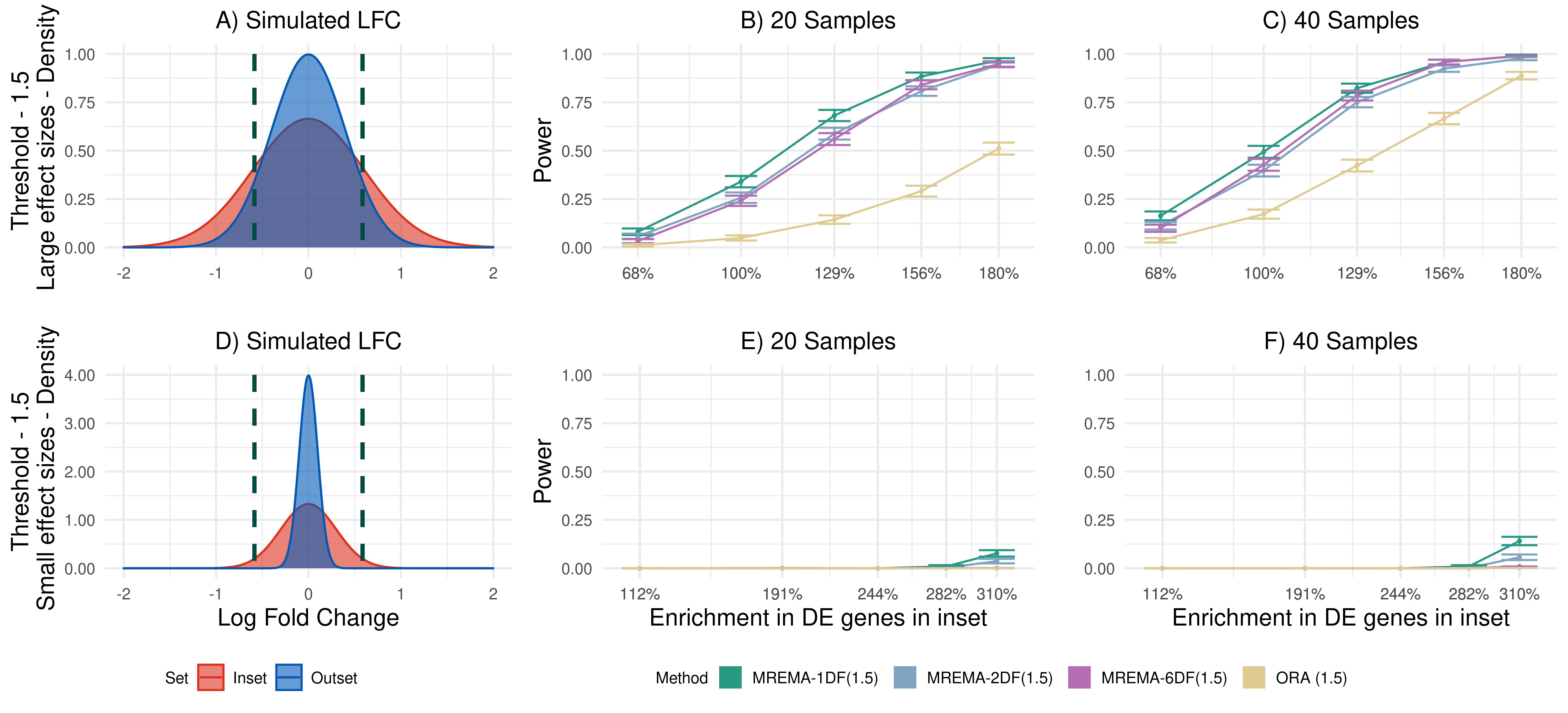

Supplement: S3 Fig — The power for the different tests is shown using a FC threshold of 1.5. The power for high LFC values illustrated in A) is shown in B) for 20 samples and C) for 40 samples. The power for low LFC values, illustrated in D) is shown in E) for 20 samples and F) for 40 samples. (TIF) [file pcbi.1010278.s003.tif]

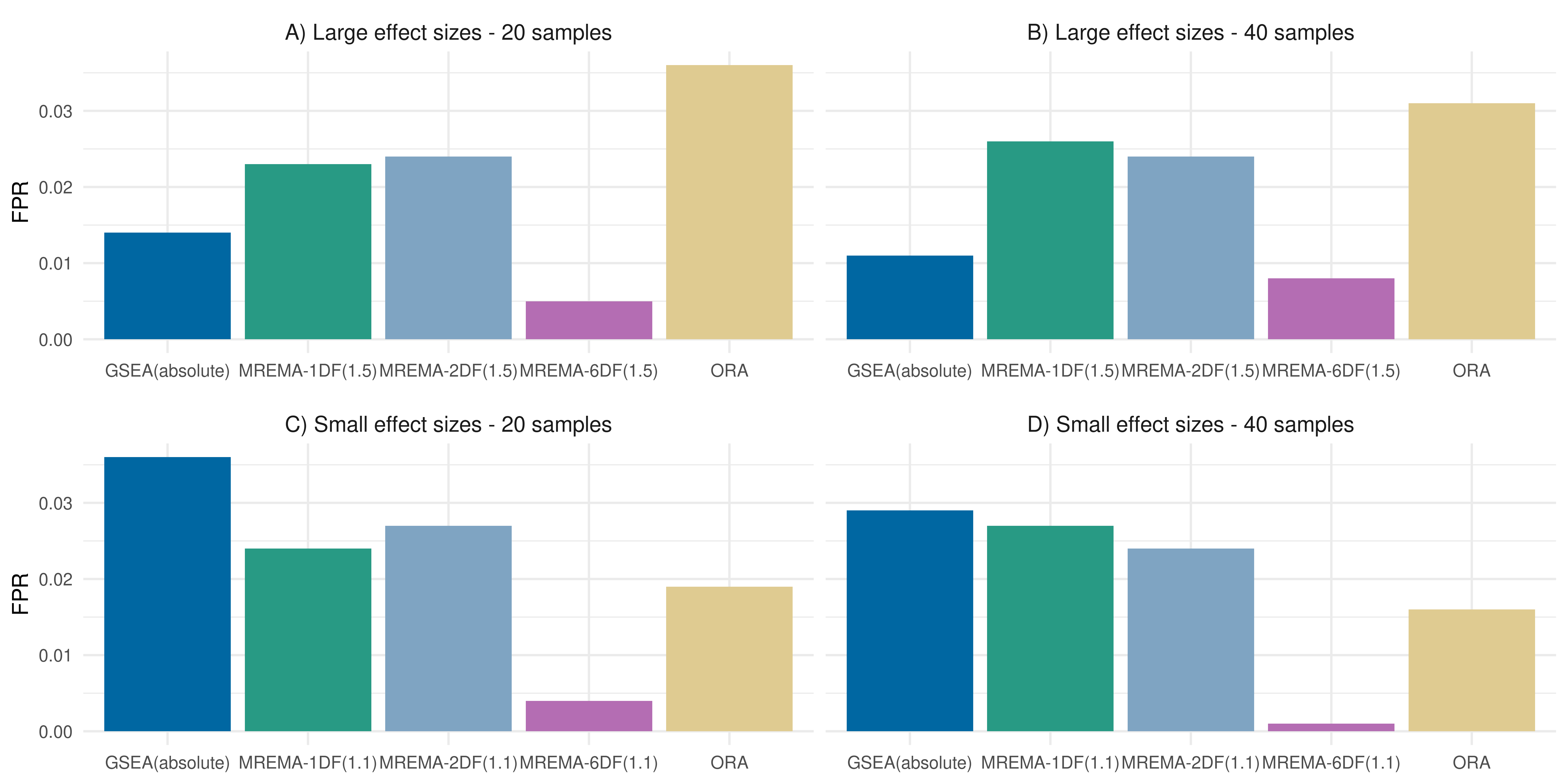

Supplement: S4 Fig — The false positive rate in null simulations with large effect sizes for A) 20 samples and B) 40 samples. The false positive rate in null simulations with small effect sizes for C) 20 samples and D) 40 samples. For our tests a gene set was deemed a false positive if the nominal p-value was less than 0.05 and the proportion of DE genes was estimated to be higher in the gene set than in the background. (TIF) [file pcbi.1010278.s004.tif]

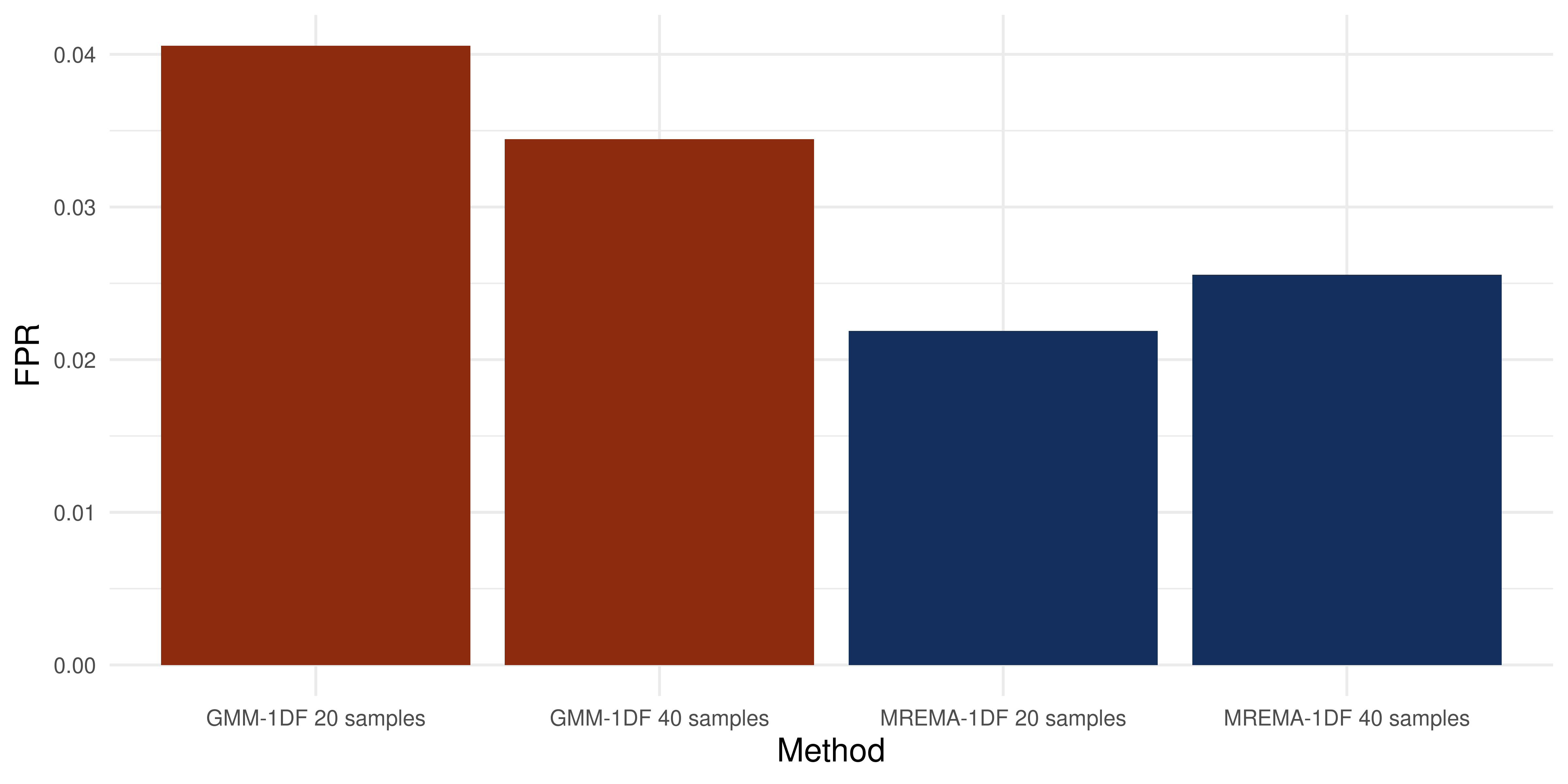

Supplement: S5 Fig — The false positive rate of the 1DF test with (blue) and without (red) accounting for the uncertainty in the LFC estimates. (TIF) [file pcbi.1010278.s005.tif]

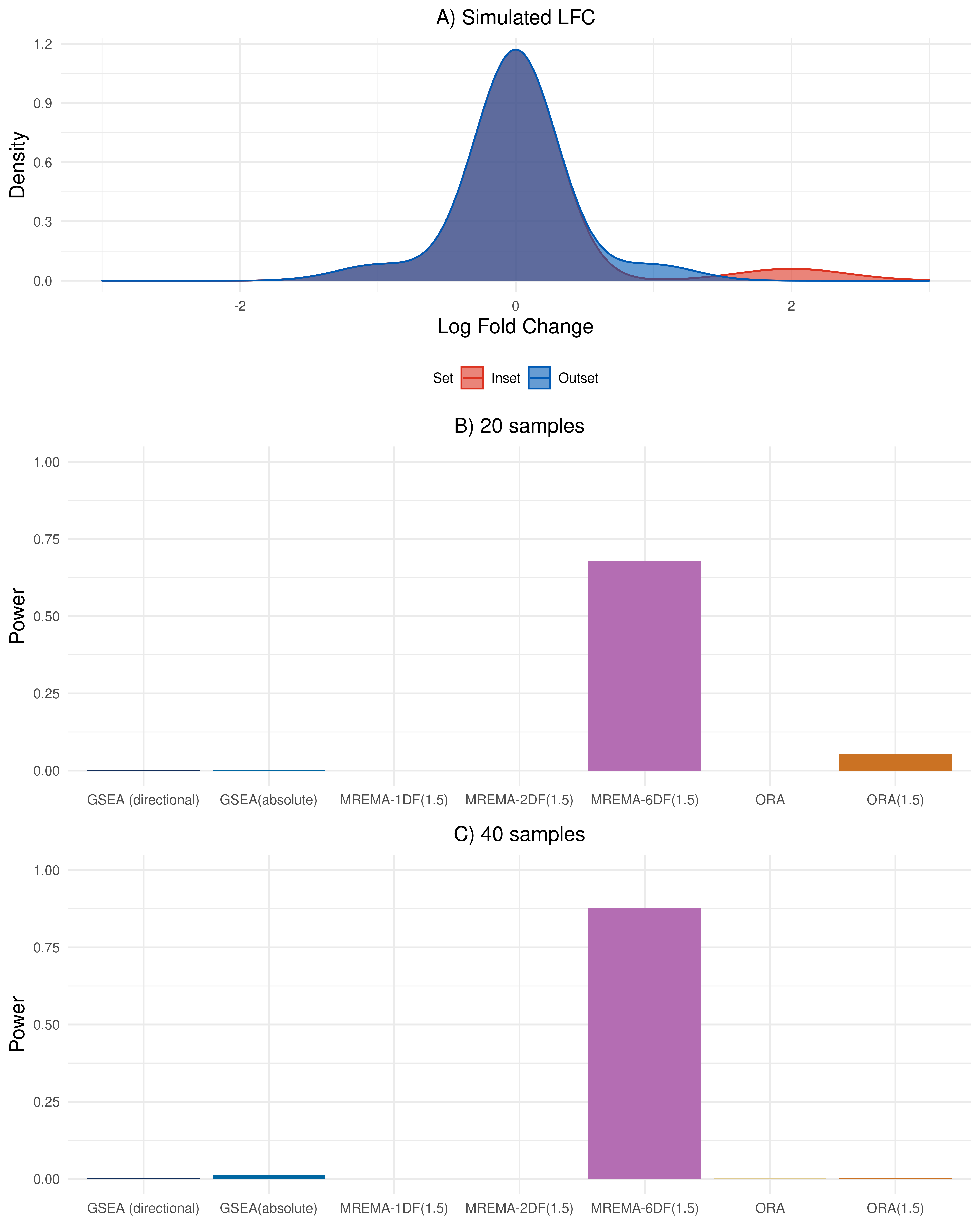

Supplement: S6 Fig — The power for all approaches as the LFC distribution changes without increasing the proportion of genes above a threshold of 1.5, for A) 20 samples and B) 40 samples. (TIF) [file pcbi.1010278.s006.tif]

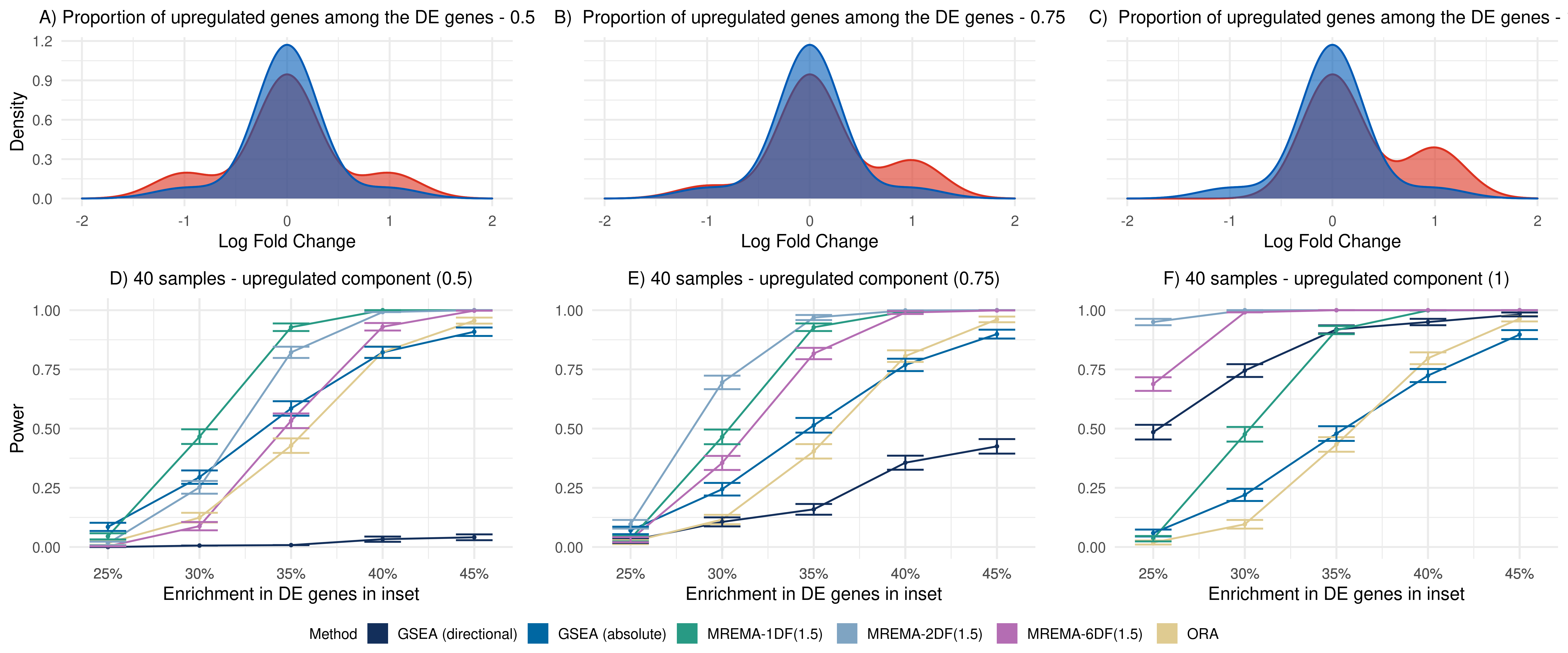

Supplement: S7 Fig — The power for all approaches when the DE genes are A & D) equally split between upregulated and downregulated, B & E) 75% upregulated and C & F) 100% upregulated. (TIF) [file pcbi.1010278.s007.tif]

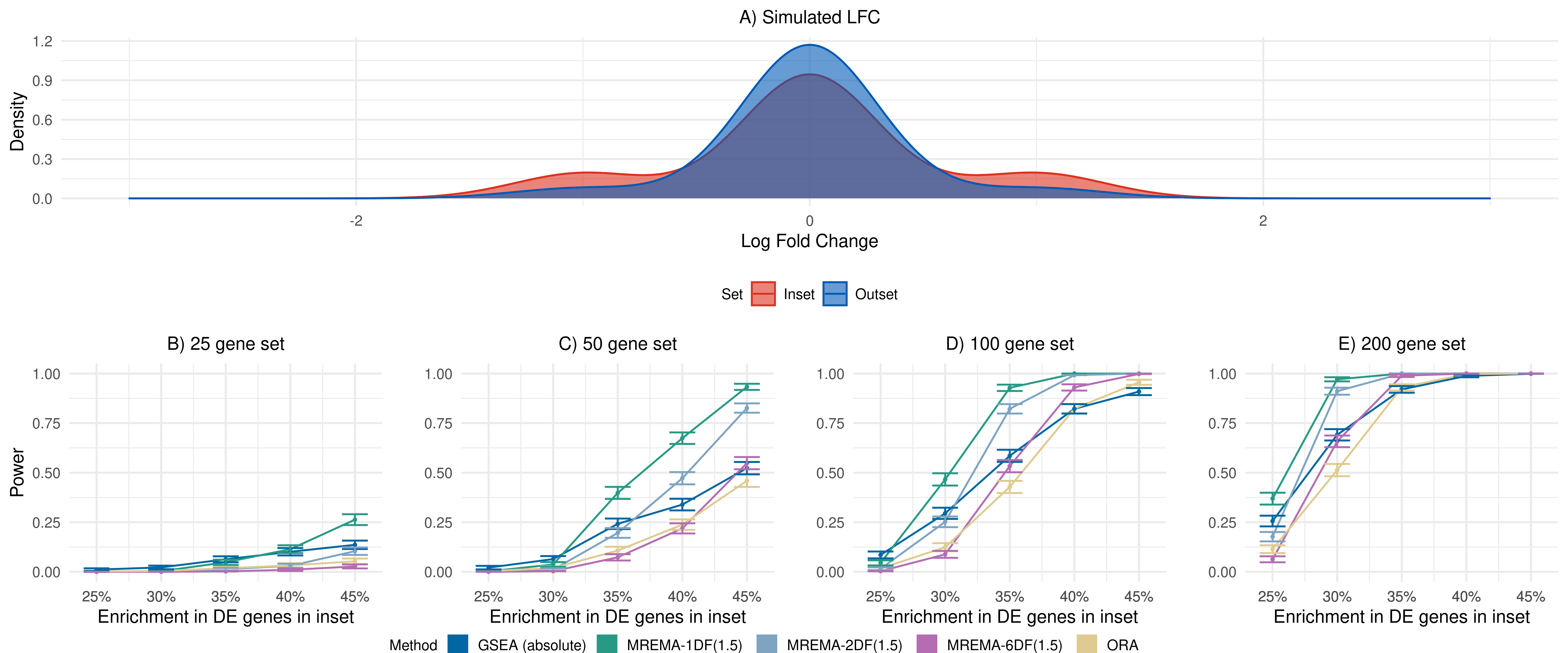

Supplement: S8 Fig — The power for all approaches to identify enrichment illustrated in red in A) as the size of the enriched gene increases from B) 25 genes to C) 50 genes to D) 100 genes to E) 200 genes. (TIF) [file pcbi.1010278.s008.tif]

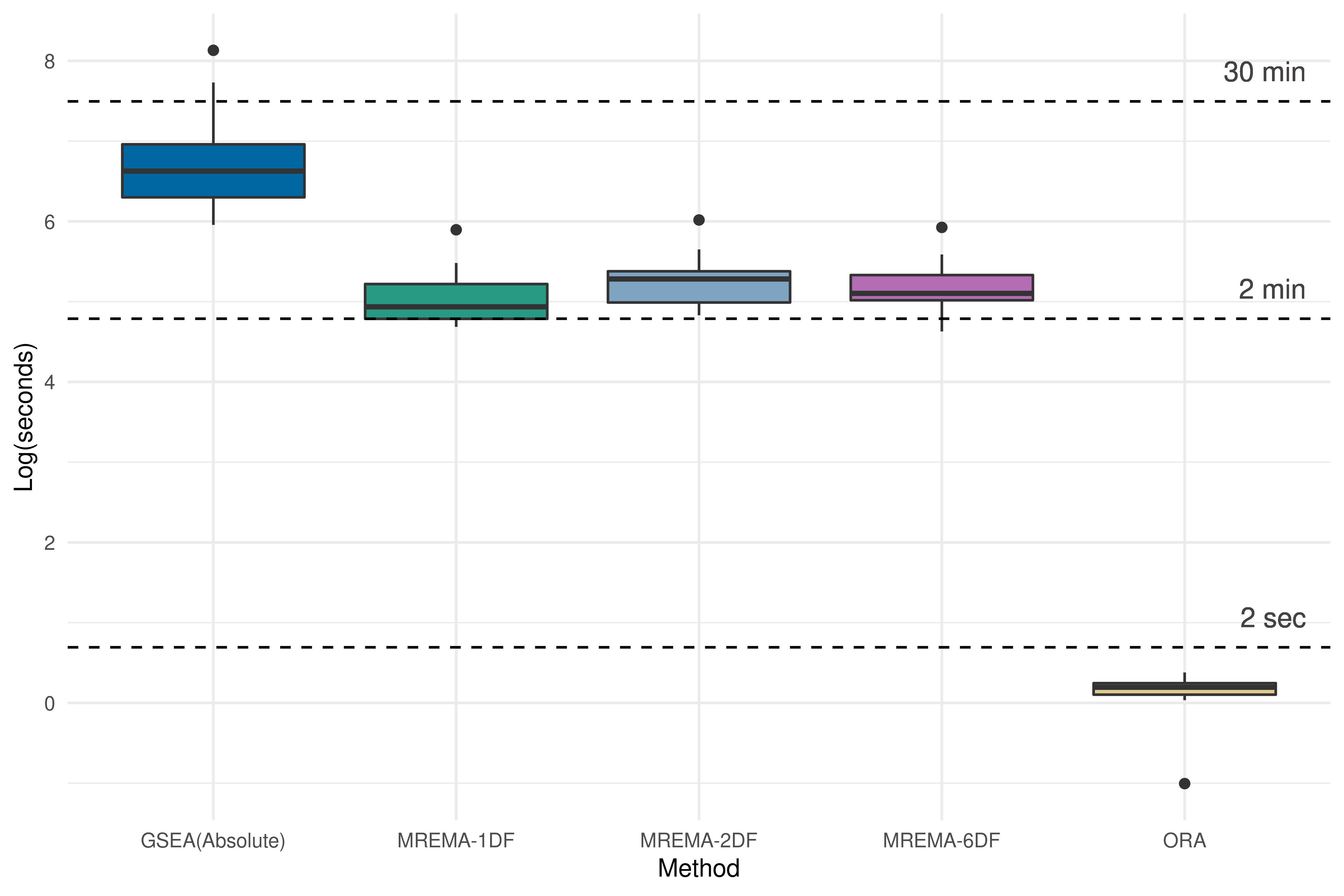

Supplement: S9 Fig — (TIF) [file pcbi.1010278.s009.tif]

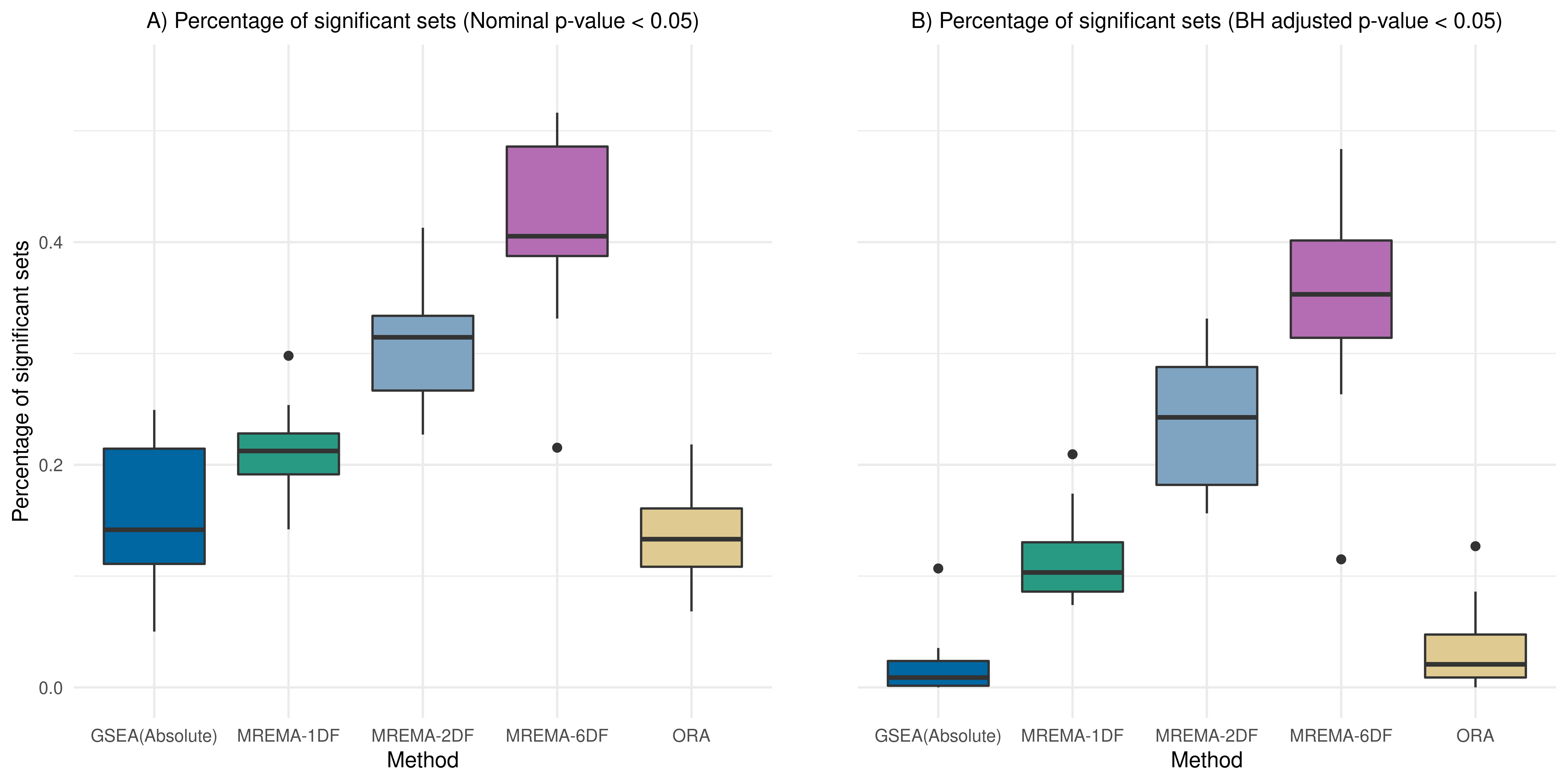

Supplement: S10 Fig — The proportion of A) nominally significant gene sets across fifteen cancer datasets and B) significant gene sets after correcting for multiple testing using the BH procedure. For our tests a gene set was deemed significant if the p-value was less than 0.05 and the proportion of DE genes was estimated to be higher in the gene set than in the background. (TIF) [file pcbi.1010278.s010.tif]

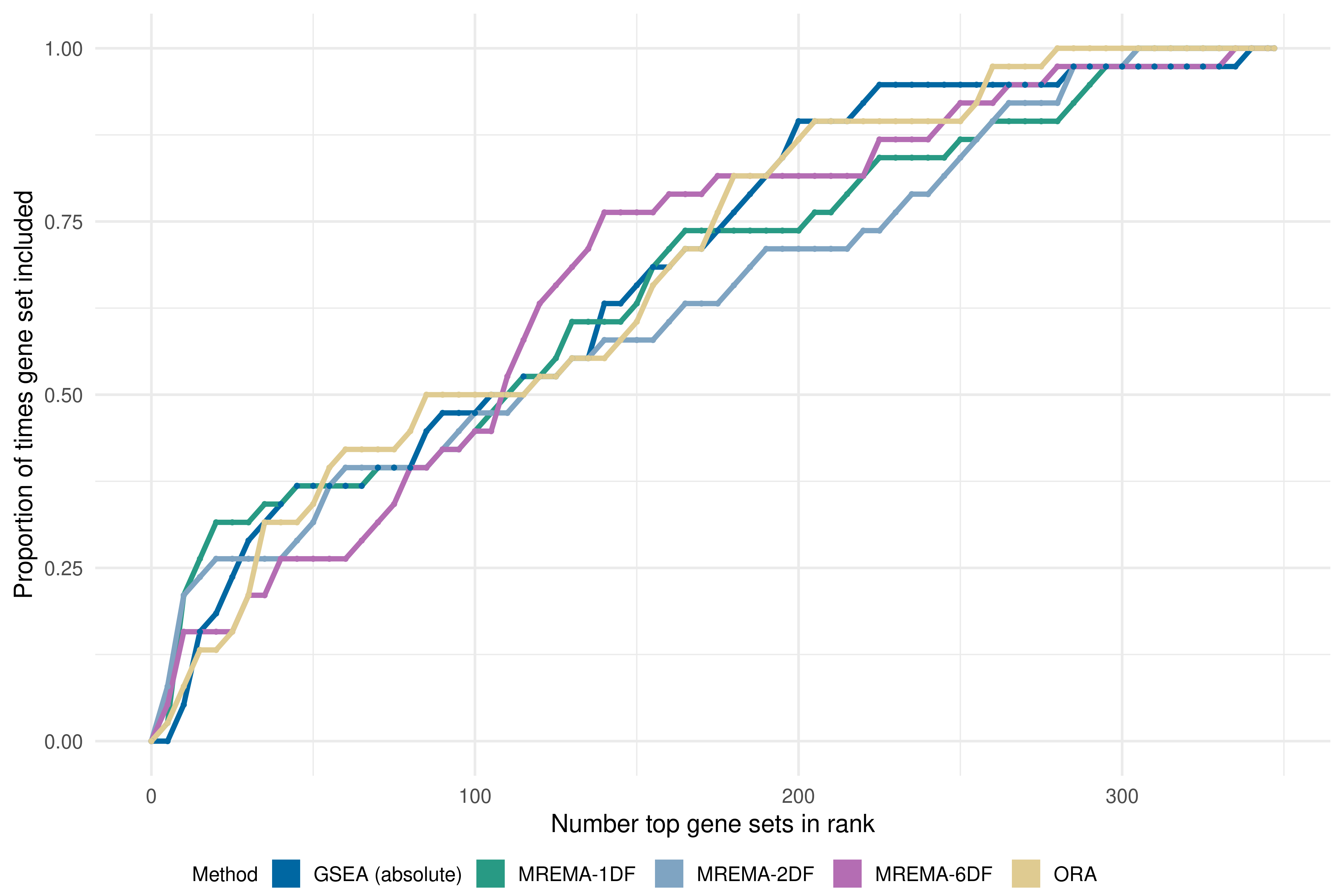

Supplement: S11 Fig — The proportion of times the disease-associated gene set was included in the x top-ranked gene sets as a function of x. This is equivalent to Fig 5, but showing the entire range of values of x. (TIF) [file pcbi.1010278.s011.tif]
