# Supplementary material for: Random-effects meta-analysis of effect sizes as a unified framework for gene set analysis
Source: PLoS Comput Biol. 2022 Oct 5;18(10):e1010278. doi: 10.1371/journal.pcbi.1010278 (PMC9576052; doi:10.1371/journal.pcbi.1010278)
Supplement: S1 Appendix — (PDF) [file pcbi.1010278.s012.pdf]

S1 Appendix - Methodology

Suppose that β_{1i} is the group effect estimated by fitting a linear regression to the data for gene i ($i = 1, \cdots, N$). We assume that β_{1i} is normally distributed with mean b_i and standard deviation equal to the standard error of the estimate of the group effect for gene i (σ_i) and that the b_i come from some parametrised distribution. We use a mixture of normal distributions, allowing a proportion of genes to be upregulated, a proportion downregulated, and a proportion that are unaffected by the group (the latter is modelled as a normal component with mean at zero and a fixed variance). The weights of the mixture component are denoted by π_l, π_2 and π_3 (corresponding to downregulated, non-DE and upregulated genes respectively), therefore the mixture distribution is represented as follows:

$$b_i \sim \sum_{l=1}^{3} \pi_l N(\mu_l, \sigma_l^2) \tag{1}$$

Therefore:

$$\beta_{1i} \sim \sum_{l=1}^{3} \pi_l N(\mu_l, \sigma_l^2 + \sigma_i^2) \tag{2}$$

We perform competitive gene set analysis by testing for differences in the distribution of the sample group effect between genes that are within a gene set compared to an appropriate background set of genes outside the gene set. Under the null model (H_0) the group effect for all genes is distributed according to (2). Under the alternative model (H_1) we have:

$$\beta_{1i} \sim \sum_{l=1}^{3} z_i \pi_{1l} N(\mu_{1l}, \sigma_{1l}^2 + \sigma_i^2) + \sum_{l=1}^{3} (1 - z_i) \pi_{2l} N(\mu_{2l}, \sigma_{2l}^2 + \sigma_i^2) \tag{3}$$

where $z_i \in \{0, 1\}$ indicates the set membership,π_{11}, π_{12} and π_{13} are the proportion of downregulated genes, non-DE genes and upregulated genes in the set respectively, likewise π_{21}, π_{22} and π_{23} are the proportion of downregulated genes, non-DE genes and upregulated genes outside of the set. $\sum_{l=1}^{3} \pi_{1l} = \sum_{l=1}^{3} \pi_{2l} = 1$, also $\mu_{12} = \mu_{22} = 0$ and σ_{12} and σ_{22} are fixed. We use the Expectation Maximization algorithm to estimate the parameters in the null and the alternative model.

The null model has 6 free parameters, $\theta = \{\mu_1, \mu_3, \sigma_1, \sigma_3, \pi_1, \pi_2\}$, the mean of the middle distribution (μ_2) is set to 0 and its variance is fixed, and the weights of the mixture components sum up to 1, leaving just two free weight parameters. In the E-step we calculate the posterior probabilities (membership probabilities) as follows: Suppose that β_{1i}, $i \in \{1, \cdots, N\}$ be our data points (i.e. inferred values of the group effect for each gene) and t_i, $i \in \{1, \cdots, N\}$ be the latent variables indicating the component from which the observation comes. Given θ, let $p(t_i = l|\beta_{1i})$ be the probability that the i-th data point comes from the l-th component given β_{1i}, then:

$$q(t_i = l) = p(t_i = l \mid \beta_{1i}) = \frac{p(t_i = l, \beta_{1i})}{p(\beta_{1i})} = \frac{p(\beta_{1i} \mid t_i = l) p(t_i = l)}{\sum_{l=1}^{3} p(\beta_{1i} \mid t_i = l) p(t_i = l)} \tag{4}$$

In the M-step, using the posterior probabilities, we estimate the parameters by maximizing the expected value of the log likelihood function:

$$\begin{aligned} Q(\theta) &= \sum_i \sum_l q(t_i = l) \log p(\beta_{1i}, t_i = l) \\ &= \sum_i [q(t_i = 1) \log p(\beta_{1i} \mid t_i = 1) p(t_i = 1) + q(t_i = 2) \log p(\beta_{1i} \mid t_i = 2) p(t_i = 2) \\ &\quad + q(t_i = 3) \log p(\beta_{1i} \mid t_i = 3) p(t_i = 3)] \end{aligned} \tag{5}$$

Considering the fact that $p(t_i = l) = \pi_l$, we have:

$$
\begin{aligned}
Q(\theta) = \sum_i \Big(& q(t_i = 1)\big(\log N(\beta_{1i} \mid \mu_1, \sigma_1^2 + \sigma_i^2) + \log \pi_1\big) + \\
& q(t_i = 2)\big(\log N(\beta_{1i} \mid \mu_2, \sigma_2^2 + \sigma_i^2) + \log \pi_2\big) + \\
& q(t_i = 3)\big(\log N(\beta_{1i} \mid \mu_3, \sigma_3^2 + \sigma_i^2) + \log \pi_3\big)\Big)
\end{aligned} \tag{6}
$$

We take partial derivatives with respect to each parameter and set it equal to zero, as this process is the same for all the mixture components, we only estimate the parameters of the first component $(\pi_1, \mu_1, \sigma_1^2)$. In order to estimate π_1 we must take into account the constraint $\sum_{l=1}^{3} \pi_l = 1$. This could be done by adding a Lagrange multiplier and maximizing the following:

$$
Q(\theta) + \lambda(\sum_{l=1}^{3} \pi_l - 1) \tag{7}
$$

Taking the partial derivative of the above function with respect to π_1 and setting it equal to 0 we get:

$$
\sum_i q(t_i = 1)\frac{1}{\pi_1} + \lambda = 0 \tag{8}
$$

Therefore:

$$
\pi_1 = \frac{\sum_i q(t_i = 1)}{-\lambda} \tag{9}
$$

Doing the same process for π_2 and π_3, we get:

$$
\pi_2 = \frac{\sum_i q(t_i = 2)}{-\lambda} \quad , \pi_3 = \frac{\sum_i q(t_i = 3)}{-\lambda} \tag{10}
$$

Summing both sides of equations in (9) and (10) and employing the constraint $\sum_{l=1}^{3} \pi_l = 1$, we find that $\lambda = -N$ where N is the total number of genes. Therefore:

$$
\pi_1 = \frac{\sum_i q(t_i = 1)}{N} \tag{11}
$$

To estimate μ_1, we take the first order partial derivative of $Q(\theta)$ with respect to μ_1:

$$
\begin{aligned}
\frac{\partial}{\partial \mu_1} Q(\theta) = \frac{\partial}{\partial \mu_1}\Big(\sum_i q(t_i = 1)\big(\log N(\beta_{1i} \mid \mu_1, \sigma_1^2 + \sigma_i^2)\Big) = \\
\frac{\partial}{\partial \mu_1}\Big(\sum_i q(t_i = 1)\big(-\frac{1}{2}\log(\sigma_1^2 + \sigma_i^2) - \frac{1}{2}\log(2\pi) - \frac{(\beta_{1i} - \mu_1)^2}{2(\sigma_1^2 + \sigma_i^2)}\Big) = \\
\sum_i q(t_i = 1)\frac{\partial}{\partial \mu_1}\Big(-\frac{(\beta_{1i} - \mu_1)^2}{2(\sigma_1^2 + \sigma_i^2)}\Big) = \\
\sum_i q(t_i = 1)\Big(\frac{(\beta_{1i} - \mu_1)}{(\sigma_1^2 + \sigma_i^2)}\Big) = 0
\end{aligned} \tag{12}
$$

Simplifying the above equation we get:

$$\sum_i q(t_i = 1)\frac{\beta_{1i}}{\sigma_1^2 + \sigma_i^2} = \sum_i q(t_i = 1)\frac{\mu_1}{\sigma_1^2 + \sigma_i^2} \tag{13}$$

We let $\omega_i = \frac{1}{\sigma_1^2 + \sigma_i^2}$ (this quantity is called the weight, corresponding to each gene), therefore we can rewrite (13) as follows:

$$\sum_i q(t_i = 1)\omega_i \beta_{1i} = \sum_i q(t_i = 1)\omega_i \mu_1 \tag{14}$$

Therefore:

$$\hat{\mu_1} = \frac{\sum_i q(t_i = 1)\omega_i \beta_{1i}}{\sum_i q(t_i = 1)\omega_i} \tag{15}$$

To estimate σ_1^2, we take the partial derivative of $Q(\theta)$ with respect to σ_1^2:

$$\begin{aligned}
\frac{\partial}{\partial \sigma_1^2} Q(\theta) = \frac{\partial}{\partial \sigma_1^2}\Bigg(\sum_i q(t_i = 1)\big(\log N(\beta_{1i} \mid \mu_1, \sigma_1^2 + \sigma_i^2)\Bigg) = \\
\frac{\partial}{\partial \sigma_1^2}\Bigg(\sum_i q(t_i = 1)\big(- \frac{1}{2}\log(\sigma_1^2 + \sigma_i^2) - \frac{1}{2}\log(2\pi) - \frac{(\beta_{1i} - \mu_1)^2}{2(\sigma_1^2 + \sigma_i^2)}\Bigg) = \\
\sum_i q(t_i = 1)\frac{\partial}{\partial \sigma_1^2}\Bigg(- \frac{1}{2}\log(\sigma_1^2 + \sigma_i^2) - \frac{(\beta_{1i} - \mu_1)^2}{2(\sigma_1^2 + \sigma_i^2)}\Bigg) = \\
\sum_i q(t_i = 1)\Bigg(\frac{-1}{2(\sigma_1^2 + \sigma_i^2)} + \frac{2(\beta_{1i} - \mu_1)^2}{4(\sigma_1^2 + \sigma_i^2)^2}\Bigg) = \\
\sum_i q(t_i = 1)\Bigg(\frac{(\beta_{1i} - \mu_1)^2 - (\sigma_1^2 + \sigma_i^2)}{2(\sigma_1^2 + \sigma_i^2)^2}\Bigg) = 0
\end{aligned} \tag{16}$$

Therefore:

$$\sum_i q(t_i = 1)\Bigg(\frac{(\beta_{1i} - \mu_1)^2 - \sigma_i^2}{(\sigma_1^2 + \sigma_i^2)^2}\Bigg) = \sum_i q(t_i = 1)\Bigg(\frac{\sigma_1^2}{(\sigma_1^2 + \sigma_i^2)^2}\Bigg) \tag{17}$$

Letting $\omega_i = \frac{1}{\sigma_1^2 + \sigma_i^2}$, we get:

$$\sum_i q(t_i = 1)\omega_i^2\Bigg((\beta_{1i} - \mu_1)^2 - \sigma_i^2\Bigg) = \sum_i q(t_i = 1)\omega_i^2 \sigma_1^2 \tag{18}$$

Therefore:

$$\sum_i q(t_i = 1)\omega_i^2 \Big((\beta_{1i} - \mu_1)^2 - \sigma_i^2\Big) = \sum_i q(t_i = 1)\omega_i^2 \sigma_1^2 \tag{19}$$

Solving the above equation we get:

$$\hat{\sigma_1^2} = \frac{\sum_i q(t_i = 1)\omega_i^2 \Big((\beta_{1i} - \mu_1)^2 - \sigma_i^2\Big)}{\sum_i q(t_i = 1)\omega_i^2} \tag{20}$$

The values of $\hat{\mu_1}$ and $\hat{\sigma_1^2}$ depend on each other, therefore we use an iterative process to calculate these quantities by first giving $\hat{\sigma_1^2}$ an initial value and iterate between them until convergence. Similarly we can estimate the other parameters in the null model (2) and finally we can get the likelihood under the null model. The alternative model in (3), has a separate set of parameters for genes inside and outside the set, hence we can estimate these parameters separately for inset and outset genes. Therefore the process is completely similar to what we did for the null model. The likelihood under the alternative model is then the sum of the likelihoods for inset and outset genes. We use likelihood ratio test to make comparison between the null and the alternative model.

We note that the alternative versions of the test are possible. In the above test, we let all the parameters of the mixture distribution (means, variances and weights) differ for genes inside and outside of a gene set (we refer to this as *MREMA-6DF*). Alternatively, if the interest is in testing whether the *proportion* of upregulated and downregulated genes inside and outside of the gene set is different without considering the magnitude of the effect, we can estimate separate weight parameters but use the same means and variances. As before we fix the variance of the middle component. We refer to this as the 2 degree of freedom test (or *MREMA-2DF*). In this case the null model is the same as the above test (2), and the alternative model is as follows:

$$\beta_{1i} \sim \sum_{l=1}^{3} z_i \pi_{1l} N(\mu_l, \sigma_l^2 + \sigma_i^2) + \sum_{l=1}^{3} (1 - z_i)\pi_{2l} N(\mu_l, \sigma_l^2 + \sigma_i^2) \tag{21}$$

where $z_i \in \{0, 1\}$ indicates the set membership, π_{11}, π_{12} and π_{13} are the proportion of downregulated genes, non-DE genes and upregulated genes in the set respectively, likewise π_{21}, π_{22} and π_{23} are the proportion of downregulated genes, non-DE genes and upregulated genes outside of the set. $\sum_{l=1}^{3} \pi_{1l} = \sum_{l=1}^{3} \pi_{2l} = 1$, also $\mu_{12} = \mu_{22} = 0$ and σ_{12} and σ_{22} are fixed. As we see, in the alternative model, the only parameters that differ between genes inside and outside of a gene set are the weights, the other parameters, means and variances of the mixture components, are shared between inset and outset genes, hence unlike the previous case, we can not estimate the parameters in the alternative model separately for inset and outset genes. In this case, we calculate the posterior probabilities in the E-step as follows:

$$q(t_i = l \mid z_i) = \frac{p(\beta_{1i} \mid t_i = l, z_i) p(t_i = l \mid z_i)}{\sum_{l=1}^{3} p(\beta_{1i} \mid t_i = l, z_i) p(t_i = l \mid z_i)} \tag{22}$$

For example, knowing that the i-th gene belongs to the set ($z_i = 1$), then the probability that it comes from the first component equals:

$$q(t_i = 1 \mid z_i = 1) = \frac{p(\beta_{1i} \mid t_i = 1, z_i = 1)\pi_{11}}{\sum_{l=1}^{3} p(\beta_{1i} \mid t_i = 1, z_i = 1)\pi_{1l}} \tag{23}$$

Also, in the M-step we maximize the following function:

$$
\begin{aligned}
Q(\theta) &= \sum_i \sum_l q(t_i = l \mid z_i) \log p(\beta_{1i}, t_i = l) \\
= \sum_i [q(t_i = 1 \mid z_i = 0) \log N(\beta_{1i} \mid \mu_1, \sigma_1^2 + \sigma_i^2)\pi_{21} &+ q(t_i = 1 \mid z_i = 1) \log N(\beta_{1i} \mid \mu_1, \sigma_1^2 + \sigma_i^2)\pi_{11} \\
+q(t_i = 2 \mid z_i = 0) \log N(\beta_{1i} \mid \mu_2, \sigma_2^2 + \sigma_i^2)\pi_{22} &+ q(t_i = 2 \mid z_i = 1) \log N(\beta_{1i} \mid \mu_2, \sigma_2^2 + \sigma_i^2)\pi_{12} \\
+q(t_i = 3 \mid z_i = 0) \log N(\beta_{1i} \mid \mu_3, \sigma_3^2 + \sigma_i^2)\pi_{23} &+ q(t_i = 3 \mid z_i = 1) \log N(\beta_{1i} \mid \mu_3, \sigma_3^2 + \sigma_i^2)\pi_{13}]
\end{aligned}
\tag{24}
$$

As in the MREMA test, we only show how to estimate the parameters of the first component (μ_1, σ_1^2). We take the partial derivative of Q with respect to μ_1 first:

$$
\begin{aligned}
&\frac{\partial}{\partial \mu_1} Q(\theta) = \\
&\frac{\partial}{\partial \mu_1}\left(\sum_i [q(t_i = 1 \mid z_i = 0) \log N(\beta_{1i} \mid \mu_1, \sigma_1^2 + \sigma_i^2) + q(t_i = 1 \mid z_i = 1) \log N(\beta_{1i} \mid \mu_1, \sigma_1^2 + \sigma_i^2)] \right) = \\
&\sum_i [q(t_i = 1 \mid z_i = 0) + q(t_i = 1 \mid z_i = 1)](\frac{\beta_{1i} - \mu_1}{\sigma_1^2 + \sigma_i^2}) = 0
\end{aligned}
\tag{25}
$$

To simplify the above equation, we let $q(t_i = 1 \mid z_i = 0) + q(t_i = 1 \mid z_i = 1) = \alpha_i$, therefore solving the above equation we get:

$$
\hat{\mu_1} = \frac{\sum_i \alpha_i \omega_i \beta_{1i}}{\sum_i \alpha_i \omega_i} \tag{26}
$$

Now, we take the partial derivative of Q with respect to σ_1^2:

$$
\begin{aligned}
&\frac{\partial}{\partial \sigma_1^2} Q(\theta) = \\
&\frac{\partial}{\partial \sigma_1^2}\left(\sum_i [q(t_i = 1 \mid z_i = 0) \log N(\beta_{1i} \mid \mu_1, \sigma_1^2 + \sigma_i^2) + q(t_i = 1 \mid z_i = 1) \log N(\beta_{1i} \mid \mu_1, \sigma_1^2 + \sigma_i^2)] \right) = \\
&\sum_i [q(t_i = 1 \mid z_i = 0) + q(t_i = 1 \mid z_i = 1)] \left(\frac{(\beta_{1i} - \mu_1)^2 - (\sigma_i^2 + \sigma_1^2)}{2(\sigma_1^2 + \sigma_i^2)^2} \right) = 0
\end{aligned}
\tag{27}
$$

Simplifying the equation we get:

$$
\hat{\sigma_1^2} = \frac{\sum_i \alpha_i \omega_i^2 \left((\beta_{1i} - \mu_1)^2 - \sigma_i^2 \right)}{\sum_i \alpha_i \omega_i^2} \tag{28}
$$

Similar to MREMA, $\hat{\mu_1}$ and $\hat{\sigma_1^2}$ are obtained in an iterative process. Also, we use LRT to compare the null and the alternative hypothesis.

A final test is possible if the interest is in testing whether the proportion of DE genes inside and outside of the gene set is different without considering the magnitude of the effect, or the split of differentially expressed genes between upregulated and downregulated. We refer to this as the 1DF of freedom test (or *MREMA-1DF*). In this case the null model is the same as the two tests above (2), and the alternative model is as follows:

$$\beta_{1i} \sim z_i\pi_1 N(\mu_1, \sigma_1^2 + \sigma_i^2) + z_i(1-\pi_1)cN(\mu_2, \sigma_2^2 + \sigma_i^2) + z_i(1-\pi_1)(1-c)N(\mu_3, \sigma_3^2 + \sigma_i^2) + (1-z_i)\pi_2 N(\mu_1, \sigma_1^2 + \sigma_i^2) + (1-z_i)(1-\pi_2)cN(\mu_2, \sigma_2^2 + \sigma_i^2) + (1-z_i)(1-\pi_2)(1-c)N(\mu_3, \sigma_3^2 + \sigma_i^2) \tag{29}$$

The means, variances and weights are estimated in that same way as in the 2DF test above. The parameter c, which controls the proportion of differentially expressed genes that are upregulated is estimated as follows:

$$c = \frac{\sum_i \Big(q(t_i = 2 \mid z_i) + q(t_i = 2 \mid (1-z_i) \Big)}{\sum_i \Big(q(t_i = 2 \mid z_i) + q(t_i = 2 \mid (1-z_i) + q(t_i = 3 \mid z_i) + q(t_i = 3 \mid (1-z_i) \Big)} \tag{30}$$
