# Supplementary material for: Random-effects meta-analysis of effect sizes as a unified framework for gene set analysis
Source: PLoS Comput Biol. 2022 Oct 5;18(10):e1010278. doi: 10.1371/journal.pcbi.1010278 (PMC9576052; doi:10.1371/journal.pcbi.1010278)
Supplement: Attachment — Submitted filename: review.pdf [file pcbi.1010278.s015.pdf]

Makrooni et al. proposed to build a gaussian mixture model (GMM) to better quantify the effect size of differentially expressed (DE) genes and the proportion of DE genes in the Gene Set Analysis (GSA). Such a GMM strategy includes an error term in estimating the Log-Fold-Change (LFC) variance for each gene to extract the LFC estimation uncertainty, boosting the gene set detection power. The authors showed the advantages of their MREMA model over the standard GSA methods through simulation and real data analysis from the TCGA datasets. Competing with the widely used GSEA method is challenging. Despite lacking a few mathematical and implementation details of the model, this paper makes me feel concerned about the persuasion to the potential users with one set of simulations (Figure1C&D) and one specific pathway detection in the TCGA-BLCA study (Figure3).

Major:

1. Model:
    a. Does i in qi represent gene index? There is no explanation on i.
    b. Does \{pi}_l in equation (1) represent the proportion of genes that are upregulated/downregulated/non-DE? Is there any constraint on \{pi}_l (what about \{pi}_1l and \{pi}_2l)? For example summation of \{pi}_l = 1?
    c. In line 327, for estimating the 1DF model, only the upregulated DE genes' proportion is estimated by all genes? What about downregulated genes' proportion?
    d. $\tau$ introduced in line 333 can also be confusing. It seems that t places constraints on the parameters of the mixture model in equations (1) and (2). Will $\tau$ be estimated iteratively in the EM algorithm? Will the DE and non-DE genes change in each iteration as $\tau$ changes? What is the relationship between $\tau$ and logFC thresholds used in Figures 1C&D and 2? How to choose the "threshold" input in the software on Github?
    e. The derivation for the p values is missing. How does the software on Github get the p values?
    f. The E-step and M-step mathematical derivations are missing. It is hard to follow equation (5) without any intermediate steps.

2. Software:
    a. What are the "mid_dist" and "size" columns? Does "mid_dist" represent over-enrich or under-enrich directionality? Maybe change it to a more meaningful name.
    b. In lines 264-266, the authors claim that ranking gene sets by the lower bound of the difference in the proportion of DE genes in the inset and outset is better than by p-value. However, the software output does not seem to provide such a ranking option. The authors also mentioned a similar concept in line 338:
    "The 95% confidence interval for the non-DE component weight was used to rank significant gene sets, with genes ranked highest having the largest difference between the lower bound of this weight in the inset and its upper bound in the outset."
    Why ranking in this way is more reasonable? Can authors intuitively interpret it?
3. The real data analysis section does not provide strong evidence of the advantage over GSEA.
    a. Using only one gene set (i.e., Calcium signaling pathway) and asking whether this particular pathway can be detected are not quite convincing for more general cases that potential users have.
    b. Besides, in S5-7 Tables, GSEA and ORA tend to have very few numbers of "significant" gene sets, and SAFE has no detection, as claimed in the paper. In practice, we may relax the adjusted p-value threshold or only consider the top 10-20 gene sets for biological interpretation. Therefore, I don't think Fig3 alone can persuade biologists to abandon the widely accepted GSEA method. Authors may consider including

researchers from the biological or biomedical field and demonstrate that the top gene set provided by MREMA is more reasonable than GSEA.

c. The example of young females with colon cancer does not add to the evidence that the MREMA model outperforms the others. Regardless of the two patients in the treatment group, there is no methods comparison. Other methods can also have similar visualization using the logFC values of each gene in the detected gene sets.

4. One set of simulations is not very convincing. Authors may consider adapting the simulation or real data analysis strategy from the comparing methods' source paper to show an advantage.
5. The information that the paragraph from lines 105 to 123 tries to convey is not very clear to me. Suppose this part aims to introduce the performance of the MREMA model under different scenarios. In that case, authors may consider including the other existing methods for comparison and organizing logically. Also, the authors may consider moving S5 Fig section to this part to show the performance of the MREMA model more comprehensively.
6. There is no implementation detail of existing methods in comparison, i.e., ORA, GSEA and SAFE. Considering there is only one gene set deemed significant per method in S5 Table and S7 Table, it is hard to decipher if the methods are properly tuned.
7. S1 Fig lacks implementation details. What does "SD0.4" mean, and what is the inset standard deviation? What is the target FDR that authors want to control? Authors may consider transforming it into similar figures in Fig1C&D to provide more comprehensive information about the false positive rate.
8. In the real data analysis, it is still unclear to me when to choose 1DF or 6DF. Should we expect to see 6DF perform better than 1DF in general, considering the parameter estimation is more sensitive. There is no evidence in the paper about the comparison of 1DF and 6DF and instructions about selection.
9. The authors mentioned several times that permutation methods in the commonly used GSA methods could be time-consuming. However, the EM algorithm that the authors proposed to estimate the parameters can also take a long time to converse. Rigorous quantification and comparison are needed to claim the runtime advantage over the existing methods.

Minor:

1. Authors may consult a professional academic writer as some sentences are very long and/or hard to follow. For example, there are two "which" in lines 247-250. What is "first stage" referring to in line 41.
2. No introduction of the full name of "GSEA."
3. S4 Fig appears in front of S2 and S3 Figs.
4. Define "inset" and "outset" when they first appear in the main text.
5. Define "power" in Fig1 & 2.
6. Fig. 3 x-axis needs more numbers to show the number of samples that each error bar corresponds to.
