# Supplementary material for: Random-effects meta-analysis of effect sizes as a unified framework for gene set analysis
Source: PLoS Comput Biol. 2022 Oct 5;18(10):e1010278. doi: 10.1371/journal.pcbi.1010278 (PMC9576052; doi:10.1371/journal.pcbi.1010278)
Supplement: Attachment — Submitted filename: Response_to_reviewers.pdf [file pcbi.1010278.s017.pdf]

Changes to manuscript

1. Supplementary appendix provided to clarify methods. Specific methodological issues raised by reviewers were also clarified within the main manuscript.
2. Ranking based on confidence intervals omitted from the revised manuscript as this does not improve performance in real data analysis and significantly increases computational cost.
3. The main simulation strategy was simplified.
4. The results for all of the MREMA tests are now shown in Results rather than just the 1DF test.
5. The figure showing the effect of accounting for the uncertainty in the LFC estimate has been moved to the Results section from the Supplement.
6. Additional simulations were added to investigate the power of different methods as the proportion of upregulated and downregulated genes changes in the enriched gene set. Another MREMA test, the 2 degree of freedom test was introduced along with these simulations.
7. Additional simulations were added to investigate the effect of varying gene set size.
8. Additional simulations provided to assess the ability of the 6DF test to identify gene sets for which the magnitude of the LFC (but not the proportion of DE genes) differs between the inset and outset.
9. Figures for simulation results were modified to improve clarity.
10. Real data analysis was revised and extended substantially.
11. Real data analysis focuses primarily on the ranking of gene sets and their nominal p-values, rather than p-values adjusted for multiple testing.
12. The effect of overlap between gene sets was included in the Discussion.
13. Recommendations are now provided in the Discussion as to which test should be used by researchers, depending on their objectives.
14. Supplementary figure added showing computational runtime.
15. Paper reviewed for overlong/unclear sentences and these were revised.
16. Definitions added to manuscript where they were previously lacking.
17. The Github page has been updated, with sample data added to allow MREMA to be run. Furthermore, the column headings in the output from our code have been improved.
18. References now use abbreviated journal names rather than the full name.
19. The order of the supplemental figures has been revised to match the order in which they appear in the manuscript.

Reviewer 1

Review of "Random-effects meta-analysis of effect sizes as a unified framework for gene set analysis"

The authors propose a novel framework for testing whether there is a difference in the distribution of differentially expressed (DE) effect sizes for the gene set of interest against the background gene set. Unlike the existing gene set analysis (GSA) methods, the proposed framework is sensitive to a wider range of signals, such as different proportion of the DE gene (1DF test), or that the proportion of DE gene is the same but the size of DE effect is different (6DF test). Specifically, the authors utilize the Gaussian mixture model (GMM) to model the effect sizes for non-DE genes and positive and negative DE genes in a given gene set. Following the random-effect meta-analysis framework, they incorporate the uncertainty of effect size estimates from each gene to increase the power of the proposed test. The Expectation-maximization algorithm is implemented to estimate the parameters in the GMM. The authors show that the proposed method is more powerful and robust than GSEA, ORA and SAFE approaches via simulation studies and real data analysis.

Overall, the manuscript is interesting and shows the improvements in power by combining the random-effect meta-analysis framework into GSA. I have several major comments/questions about the manuscript for the authors:

1. It would be easier for readers to understand/follow the proposed framework if the authors provide more details about the methodology in the Methods section or in the supplementary materials. The following are some examples that can be elaborated on for clarity:

   Response: We thank the reviewer for raising this point. A detailed methodology document has been provided and added to our supplementary materials (referred to here as S1 appendix).

   (a) In GMM subsection, it is unclear of what the weight π_l means here. Can the weight $\pi_l, l = 1,2,3$ be viewed as the proportional of non-DE, up-regulated, and down-regulated DE genes in the gene set? Or is this weight π_l equivalent to weight adjusting for uncertainty in each gene (Eq. 5), $W_i = \frac{1}{\sigma_i^2+\sigma_l^2}$.

   Response: $\pi_l, l = 1,2,3$ *are the weights of the components in the mixture distribution, hence they indicate the proportion of non-DE, up-regulated and down-regulated genes. These are defined more clearly in the supplementary material of the revised manuscript. [S1 Appendix]*

   (b) Does the model parameters set include $\theta_0 = (\pi_l, \mu_l, \sigma_l), l = 1,2,3$ (under the null hypothesis) and $\theta = (\pi_{1l}, \mu_{1l}, \sigma_{1l}, \pi_{2l}, \mu_{2l}, \sigma_{2l}), l = 1,2,3$ (under the alternative hypothesis) that need to be estimated? Is σ_i^2 the input data and viewed as fixed value? Assume there are m_1 genes in the inset and m_2 genes in the outset, does the algorithm only use the effect size estimates data (or LFC) from these m_1 genes to estimate $(\pi_{1l}, \mu_{1l}, \sigma_{1l})$?

Response: Yes, σ_i^2 is the input data. It is the uncertainty in the LFC estimate. As described in the S1 Appendix, LFC and σ_i^2 are estimated by fitting a linear regression to the gene expression data. We have defined different tests, with different alternative hypotheses. Depending on the test, some of these parameters may be shared between the inset and outset genes. For example, in the 6DF MREMA test, as described in the S1 Appendix (equation 2 and 3) there are 6 free parameters under the null model $\theta_0 = (\pi_1, \pi_2, \mu_1, \mu_3, \sigma_1, \sigma_3)$,. Note that the mean and the variance of the middle distribution are fixed. In the alternative model, we have separate parameters for genes inside and outside of the gene set, hence the set of free parameters in the alternative model is $\theta = (\pi_{11}, \pi_{12}, \mu_{11}, \mu_{13}, \sigma_{11}, \sigma_{13}, \pi_{21}, \pi_{22}, \mu_{21}, \mu_{23}, \sigma_{21}, \sigma_{23})$. In this case the Reviewer is correct and the estimation procedure is equivalent to using m_1 genes to estimate the inset parameters and m_2 genes to estimate the outset parameters (with m_1 and m_2 as defined by the Reviewer). However, in cases in which the inset and outset have some shared parameters, we cannot estimate the parameters separately in the two groups of genes.

(c) In lines 327-329, it says "the weights assigned to the non-DE genes …". Is the same estimation procedure conducted for the positive/negative DE in the inset/outset?

Response: As described in equation (29) in the S1 Appendix, the weight parameter for the non-DE genes is not a shared parameter for genes inside and outside of the gene set, hence they could be estimated separately, using only genes inside the gene set to estimate the weight of non-DE genes in the set, and genes outside of the set to estimate the weight parameter of the non-DE genes outside of the set. However, the parameter c in this equation (the proportion of up-regulated genes among DE genes) is a shared parameter between the inset and outset genes (under the alternative model), hence the estimation procedure considers all genes together.

(d) The implementation of EM algorithm is not clear. It would be very helpful if the authors provide details about their use of the EM algorithm or some pseudo-code to help readers better understand and use their framework.

Response: The details of the EM algorithm for different versions of our model are provided in the S1 Appendix included with the revised manuscript.

(e) It is unclear how to place the constraint on the parameters of GMM (lines 333-335). The reasons on how to set this constraint are not elaborated and the choice of values of 0.25/0.95 seems arbitrary.

Response: This information is provided in the Methods section of the revised manuscript. Briefly, for each iteration of the EM algorithm, in the M-step, a maximum/minimum allowable value for the mean of the DE component is calculated, given the new estimate for variance. If the new estimate for the mean then exceeds this allowable value, the max/min allowable value is used in its place. The 0.95 constraint for non-DE component was chosen to allow only a small proportion of the non-DE component to exist outside of the DE thresholds. A more relaxed constraint (a minimum of 0.75 of the weight of the DE components beyond the thresholds, corresponding to a maximum of 0.25 of the weight within the thresholds)

was used for the DE components. This more relaxed constraint allows the variance of the DE components to be larger, which helps to prevent outliers, with very large values of the LFC, having a very large impact on the means of the DE components. In simulations we have found that our method is not very sensitive to the values used to define these soft thresholds.

(f) It would be helpful if the authors can give more details about hypothesis testing. The authors use EM algorithm to estimate the parameters in GMM after incorporating the with-gene uncertainty. However, it is unclear what statistical test is conducted in the 1DF or 6DF test, and what the corresponding test statistics are. From lines 64-66, it seems that the likelihood ratio test is used, but it is not stated explicitly. Moreover, in line 158, the authors only report a p-value of "P=9.2×10^{-60}" but give no mention of what statistical test was used.

Response: The statistical test we used is the Likelihood Ratio Test. We apologies that this was not clear in the original manuscript, details have been added to the section Model and Implementation.

(g) Similarly, for the 95% confidence interval on the proportion of DE genes (line 74-75), how was it constructed, especially when then sample size is quite small (n=5~40)?

Response: We have removed the ranking of gene sets based on the confidence intervals from the manuscript. We found that ranking based on the confidence intervals did not work well on real data and it comes at substantial computational cost. The large CIs for parameters estimated in small gene sets, highlighted by the Reviewer, may be the reason that the use of the CIs for gene set ranking appears not to be beneficial.

2. One of the more major innovations of the proposed framework is that it can identify differences in the mixture distribution between the inset and outset. However, this claim is not supported by any simulation studies or real data set. The performance (such as power and false positive rate) of the proposed 6DF test is unknown. Additional simulation studies should be considered and presented, where the only differences are in the signs or the sizes of the DE effects while the proportion of DE genes in the inset/outset are the same. Such a simulation study provides evidence on how the 6DF test identifies gene sets with differences in distribution in DE effects and what gain exists in using the proposed framework over the 1DF test and other existing methods.

   Response: For the revised manuscript we have added the results for the 6DF test to the figures for the power and false positive rate. We have also added new simulation scenarios in which the proportions of up-regulated/down-regulated genes differ between the inset and outset (supplementary figure 7) and in which the effect sizes change without any change in the proportion of DE genes (supplementary figure 6). The latter illustrates that the 6DF method can identify differences between the inset and outset that other methods are not designed to detect.

3. The proposed method requires a pre-specified threshold τ to define DE (line 333). The choice of τ seems to impact the power for the proposed method (Fig 2) and ORA (S4 Fig) largely when

there are different standard deviations between inset and outset. It would be helpful if authors provide more guideline of how to choose this value in practice since the true distribution of inset/outset is unknown. Would it further increase power if the proposed method used a grid search to find the optimal threshold τ, a common practice implemented in SKAT-O (Lee et al 2012)?

Lee, S., Emond, M.J., Bamshad, M.J., Barnes, K.C., Rieder, M.J., Nickerson, D.A., Team, E.L.P., Christiani, D.C., Wurfel, M.M., Lin, X. and NHLBI GO Exome Sequencing Project, 2012. Optimal unified approach for rare-variant association testing with application to small-sample case-control whole-exome sequencing studies. The American Journal of Human Genetics, 91(2), pp.224-237.

Response: The choice of the pre-specified threshold does affect the results, as highlighted by the Reviewer. We note that this threshold is analogous to thresholds that are often applied in gene expression analysis, including in the context of GSA. For example, ORA can be used to test for enrichment of significantly DE genes with absolute log fold threshold; however, not specifying thresholds in this way runs the risk of resulting in an ill-defined test with undesirable and unpredictable behaviours with changing sample size. This is because the proportions of genes that are statistically significantly DE in the inset and outset depends on the sample number as well as the distribution of effect sizes in the inset and outset (e.g. a gene set that is significantly enriched when a given number of samples is analyzed may be no longer enriched when the number of samples increases if the background gene set contains more genes with modest effect sizes). Forcing the user to specify a DE threshold avoids these anomalies, resulting in more consistent results. As in any statistical test, it is critical to specify the hypothesis before considering the data. Because the threshold used is an aspect of the hypothesis being tested, we do not consider that it would be correct to perform a grid search for an optimal threshold.

Other minor comments/questions about the manuscript are listed below:

1. In the introduction, since the authors mention some ranking-based approaches may be computationally expensive, the paper would benefit from the authors sharing comparison results on the computational time of the proposed method with existing approaches.

   Response: This has been included in supplementary figure 9. We thank the reviewer for their suggestion.

2. Some sentences are too long and wordy putting an unnecessary burden on readers. For example, the sentence in lines 359-363 may be easier to follow if it was split into two or more clear and concise sentences.

   Response: We have amended the sentence highlighted by the reviewer and have reviewed the manuscript with a view to avoiding overly long sentences.

3. The legend of supplementary tables should be more specific. For example, in S1 Table "Gene sets identified by 1DT test with a threshold of 1.5". It's not clear to know what the threshold is about without referring to the main text.

   Response: The reviewer is correct. The figures have been divided so that the results for only one threshold are shown in each figure, and additional detail has been provided in the figure captions, in order to clarify this.

4. The order of figures is not in reading order, e.g., the main text mentions S4 Fig before S2/S3 Fig. Also, Fig 1B is not mentioned by the main text. It's not clear how the proportion DE is calculated in the Fig 1B table. Is it the average empirical proportion of DE genes in the simulation studies or is it the area under the curve for the tail of the normal distribution?

   Response: The proportion of the DE is the average empirical proportion of DE genes in the simulation studies. We have removed the table in Fig 1B, and instead replaced the x-axis for the power plots with the percentage enrichment in upregulated genes in the inset over the outset. This removes the need for consulting the table with each increase in standard deviation. We thank the reviewer for highlighting the difficulty in interpreting the original figures. We have reviewed the manuscript and correctly named our supplementary figures.

5. Legend is inconsistent and mislabeled. For example, the proposed method is named as 1DFT1.5 in the Fig 1, while in S2 Fig and S3 Fig, it's named as T1.5-DF7.

   Response: This has been amended. We thank the reviewer for highlighting this error.

6. How many genes were generated in the RNA-Seq gene expression data? What's the size of each non-overlapping gene sets? Also, how was the RNA-Seq data used in the simulation (since later LFC was generated from normal distribution)?

   Response: Expression values for 10,000 genes were simulated in each simulation. In the main power simulations, Fig.1, Fig.2 and Fig.3, 100 of these genes were in the enriched gene set and 9,900 genes were divided into 99 non-enriched gene sets of the same size. The count data was simulated using the negative binomial distribution while the normal distribution was used to simulate the LFC distribution in Fig.1, Fig.2 and Fig3.

7. It is unclear what the LFC power simulation settings are (lines 378-380). In this setting, each power simulation contains one truly enriched gene set (with 100 genes) and the outset contains 9900 genes and is repeated 1000 times. So, will the power simulation return 1000 p-values in total? Where does the 9000 non-enriched sets p-values come from? It will also help readers to understand if authors would specify explicitly how they calculate the power and false positive rate for each approach in the simulation studies.

   Response: The simulation design has been simplified for ease of communication. In each power simulation there is now one enriched gene set and 99 non-enriched gene sets, each with 100 genes. This returns 100 p-values which were adjusted for multiple testing. If our approach returned an adjusted p-value below 0.05 and the estimated proportion of DE genes was higher in the enriched set than in the background, it was deemed to have successfully identified the enriched set. This procedure was repeated 1,000 times to estimate power. For the null simulations, 100 non-enriched sets were tested for enrichment and any set with a nominal p-value below 0.05 was deemed to be a false positive. This was repeated 10 times to give 1,000 p-values for non-enriched sets.

8. In TCGA data analysis, was group membership included as an interaction term when estimating the fold change between different patients (lines 398-399)?

Response: Group membership was included as an interaction term for the analysis; however, this section of the manuscript has been replaced by a more comprehensive analysis of real data at the suggestion of other reviewers.

9. For the R code on Github, the example data set is not available and so the example code cannot be run.

   Response: We have included an example data set.

10. PLOS Computational Biology uses ICMJE reference style, and the authors should use the abbreviation instead of full name of the journal, e.g., Genet Mol Res instead of Genetics and Molecular Research.

    Response: We thank the reviewer for highlighting this.

Reviewer 2

Makrooni et al. proposed to build a gaussian mixture model (GMM) to better quantify the effect size of differentially expressed (DE) genes and the proportion of DE genes in the Gene Set Analysis (GSA). Such a GMM strategy includes an error term in estimating the Log-Fold-Change (LFC) variance for each gene to extract the LFC estimation uncertainty, boosting the gene set detection power. The authors showed the advantages of their MREMA model over the standard GSA methods through simulation and real data analysis from the TCGA datasets. Competing with the widely used GSEA method is challenging. Despite lacking a few mathematical and implementation details of the model, this paper makes me feel concerned about the persuasion to the potential users with one set of simulations (Figure1C&D) and one specific pathway detection in the TCGA-BLCA study (Figure3).

Response: We have extended the simulations and real data analysis, as described in responses below to the Reviewer's comments.

Major:
1. Model:

A. Does i in qi represent gene index? There is no explanation on i.

   Response: Yes, i in qi represents the gene index. This has been explained in the revised manuscript.

B. Does \{pi}_l in equation (1) represent the proportion of genes that are upregulated /downregulated /non-DE? Is there any constraint on \{pi}_l (what about \{pi}_1l and \{pi}_2l)? For example summation of \{pi}_l = 1?

   Response: In equation (1), $\pi_l, l = 1,2,3$ are the proportion of genes that are up-regulated/down-regulated and non-DE. These are the weights of the components in the mixture distribution, hence they are required to add up to 1. This constraint has been stated in the revised manuscript. There is the same constraint on the weights of the mixture components for genes inside and outside of the set. For example, in equation (2), $\pi_{1l}, l = 1,2,3$ add up to 1 also $\pi_{2l}, l = 1,2,3$ add up to 1. A detailed description of the model is provided in the supplementary material of the revised manuscript [S1 Appendix].

C. In line 327, for estimating the 1DF model, only the upregulated DE genes' proportion is estimated by all genes? What about downregulated genes' proportion?

   Response: This line referred to the proportion of DE genes that are upregulated. The proportion of DE genes that are downregulated is one minus this. The extended description of the methods (provided as S1 Appendix with the revised manuscript) provides greater clarity. As described in this appendix, in the 1DF test, the proportion of DE genes that are up-regulated is denoted by c, which is estimated using equation (30) of the S1 Appendix. The proportion of the down-regulated genes is then 1-c.

D. t introduced in line 333 can also be confusing. It seems that t places constraints on the parameters of the mixture model in equations (1) and (2). Will t be estimated iteratively in the EM algorithm? Will the DE and non-DE genes change in each iteration as t changes? What is the relationship between t and logFC thresholds used in Figures 1C&D and 2? How to choose the "threshold" input in the software on Github?

Response: Tau and the logFC thresholds in Fig.1 and Fig.2 are the same. This does place constraints on the mixture model in that it forces the mean to remain above the positive threshold for the upregulated component and below the negative threshold for the negative component. For each iteration of the EM algorithm, in the M-step, a maximum/minimum allowable value for the DE component means is calculated with the new estimate for variance. If the new estimate for the mean then exceeds this allowable value, the max/min allowable value is used in its place. The threshold parameter in the function on github allows the user to provide a fold change threshold above which the researcher considers a gene to be DE. Additional detail has been provided in the methods section on line 353-363.

E. The derivation for the p values is missing. How does the software on Github get the p values?

Response: The p-value is derived using the LRT on lines 48, 85 and 109 of the mrema.R script on Github. In the revised manuscript we state that the p-values are obtained from the LRT.

F. The E-step and M-step mathematical derivations are missing. It is hard to follow equation (5) without any intermediate steps.

Response: We thank the reviewer for raising this point. A detailed methodology document has been provided and added to our supplementary materials for the revised manuscript.

.

2. Software:

A. What are the "mid_dist" and "size" columns? Does "mid_dist" represent over-enrich or under-enrich directionality? Maybe change it to a more meaningful name.

Response: "mid_dist" indicates whether the proportion of DE genes was estimated to be higher in the inset than the outset. We have replaced the column name with "Prop.DE.Increased". "size" is the number of genes in a set and this column name has been replaced with "NR.GENES". We acknowledge that the previous titles were confusing and thank the reviewer for their suggestion.

B. In lines 264-266, the authors claim that ranking gene sets by the lower bound of the difference in the proportion of DE genes in the inset and outset is better than by p-value. However, the software output does not seem to provide such a ranking option. The authors also mentioned a similar concept in line 338: "The 95% confidence interval for the non-DE component weight was used to rank significant gene sets, with genes ranked highest having the largest difference between the lower bound of this weight in the inset and its upper bound in the outset."Why ranking in this way is more reasonable? Can authors intuitively interpret it?

Response: A more comprehensive analysis of real data (which we carried out in response to the Reviewer's point 3, below) gave poor results when genes were ranked using the confidence intervals, as discussed above, in response to Reviewer 1 point 1(g). This may be a consequence of

wide confidence intervals for small gene sets. Consequently, we have removed this section from the manuscript and report only the ranking based on statistical significance, which is more directly comparable in any case to the rankings of gene sets produced by other methods.

3. The real data analysis section does not provide strong evidence of the advantage over GSEA.

   A. Using only one gene set (i.e., Calcium signaling pathway) and asking whether this particular pathway can be detected are not quite convincing for more general cases that potential users have.

   Response: *We accept the Reviewer's point and, in response, have carried out a much more comprehensive evaluation of method performance on real data. This consisted of an analysis of fifteen different cancer datasets in addition to a dataset of multiple human diseases used by Tarca et al., 2012 when introducing PADOG (Fig.4 and Fig.5 of the revised manuscript). The results obtained in this more comprehensive evaluation provide support for an advantage of our method over GSEA in many cases.*

   Tarca AL, Draghici S, Bhatti G, Romero R. Down-weighting overlapping genes improves gene set analysis. BMC Bioinformatics. 2012;13(1). doi:10.1186/1471-2105-13-136.

   B. Besides, in S5-7 Tables, GSEA and ORA tend to have very few numbers of "significant" gene sets, and SAFE has no detection, as claimed in the paper. In practice, we may relax the adjusted p-value threshold or only consider the top 10-20 gene sets for biological interpretation. Therefore, I don't think Fig3 alone can persuade biologists to abandon the widely accepted GSEA method. Authors may consider including researchers from the biological or biomedical field and demonstrate that the top gene set provided by MREMA is more reasonable than GSEA.

   Response: *We accept the Reviewer's point that the adjusted p-value threshold may be overly stringent and, consequently, we compared our method with established methods using nominal p-values and by evaluating the ranking of gene sets. These results are shown in Fig.4 and Fig.5 of the revised manuscript.*

   C. The example of young females with colon cancer does not add to the evidence that the MREMA model outperforms the others. Regardless of the two patients in the treatment group, there is no methods comparison. Other methods can also have similar visualization using the logFC values of each gene in the detected gene sets.

   Response: *The Reviewer is correct that the comparison of the group effect between sample subgroups is not something that could only be achieved using our method. This section has been removed from the manuscript. However, while it is possible to visualize LFC values without our method, visualizing the parametrized inset and outset distributions as estimated by our method provides insights as to the reason for the rejection of the null hypothesis for a given gene set that is deemed to be enriched. Furthermore, the distribution estimated by our method takes into account the uncertainty in the LFC values for individual genes.*

4. One set of simulations is not very convincing. Authors may consider adapting the simulation or real data analysis strategy from the comparing methods' source paper to show an advantage.

Response: *Additional sets of simulations have been added in supplementary figure 6, 7, and 8. Real data analysis strategies have been adapted from GSEABenchmarkeR and Tarca et al., 2012. We thank the reviewer for suggesting these improvements.*

Tarca AL, Draghici S, Bhatti G, Romero R. Down-weighting overlapping genes improves gene set analysis. BMC Bioinformatics. 2012;13(1). doi:10.1186/1471-2105-13-136.

5. The information that the paragraph from lines 105 to 123 tries to convey is not very clear to me. Suppose this part aims to introduce the performance of the MREMA model under different scenarios. In that case, authors may consider including the other existing methods for comparison and organizing logically. Also, the authors may consider moving S5 Fig section to this part to show the performance of the MREMA model more comprehensively.

Response: *Fig. S5 has been moved to the main results as suggested. We have amended the paragraph highlighted in order to help improve clarity. The paragraph seeks to highlight, as the reviewer suggests, the performance of MREMA using different thresholds under different scenarios. For example, if the LFC values are low a high threshold will fail to identify any enriched gene sets as the regions between the negative and positive threshold may have weight for the inset and outset. An example of this can be seen in S3 Fig D, E, and F, where a threshold of 1.5 suggests very little difference between the enriched set and the non-enriched sets.*

6. There is no implementation detail of existing methods in comparison, i.e., ORA, GSEA and SAFE. Considering there is only one gene set deemed significant per method in S5 Table and S7 Table, it is hard to decipher if the methods are properly tuned.

Response: *Additional detail has been provided in the methods section. As previously mentioned by the Reviewer the multiple testing correction was very stringent on existing methods and as such is not carried out in real data analysis.*

7. S1 Fig lacks implementation details. What does "SD0.4" mean, and what is the inset standard deviation? What is the target FDR that authors want to control? Authors may consider transforming it into similar figures in Fig1C&D to provide more comprehensive information about the false positive rate.

Response: *S1 Fig has been amended and is now Fig S4.. SD0.4 refers to the standard deviation of the LFC distribution of all gene sets in the null simulations. This has been replaced by the subtitles "Large effect sizes" and "Small effect sizes" corresponding to Fig.1 and Fig.2 respectively. In the Null simulations there are no enriched gene sets, as such, mimicking Fig.1 and Fig.2 exactly is not possible.*

8. In the real data analysis, it is still unclear to me when to choose 1DF or 6DF. Should we expect to see 6DF perform better than 1DF in general, considering the parameter estimation is more sensitive. There is no evidence in the paper about the comparison of 1DF and 6DF and instructions about selection.

Response: *The results from the 6DF test have been included in the manuscript. We have also included a section in the discussion, on lines 308-316, providing guidelines for test selection. We would not expect the 6DF test to always perform better. For example if the inset has a higher proportion of DE genes than the background genes but the effect sizes of the DE genes are similar in the inset and outset, then the 6DF test should have less power to detect this difference than the 1DF or 2DF tests. This is because the*

additional degrees of freedom for the likelihood ratio test in the 6DF test results in a decrease in power, when these additional degrees of freedom do not contribute to an improvement in model fit.

9. The authors mentioned several times that permutation methods in the commonly used GSA methods could be time-consuming. However, the EM algorithm that the authors proposed to estimate the parameters can also take a long time to converse. Rigorous quantification and comparison are needed to claim the runtime advantage over the existing methods.

Response: *This has been included in supplementary figure 9. We thank the Reviewer for this suggestion.*

Minor:

1. Authors may consult a professional academic writer as some sentences are very long and/or hard to follow. For example, there are two “which” in lines 247-250. What is “first stage” referring to in line 41.

   Response: *We have reviewed the manuscript for clarity. The line mentioned above has been amended and can be found on line 268 of the new manuscript. “First stage” refers to the differential expression analysis part of an analysis. This term has been replaced with a more explicit reference to the procedure.*

2. No introduction of the full name of “GSEA.”

   Response: *This has been included on line 24 with thanks to the reviewer for pointing out this oversight.*

3. S4 Fig appears in front of S2 and S3 Figs.

   Response: *The names of the supplementary figures have been changed to correspond to their order in the manuscript.*

4. Define “inset” and “outset” when they first appear in the main text.

   Response: *These definitions have been added to the manuscript on line 15 with thanks to the reviewer for pointing out this oversight.*

5. Define “power” in Fig1 & 2.

   Response: *The power is defined as the proportion of times that the enriched gene sets is identified as significantly enriched, this clarification has been added to the manuscript on line 102 of the revised manuscript.*

6. Fig. 3 x-axis needs more numbers to show the number of samples that each error bar corresponds to.

   Response: *Fig.3 is no longer part of the real data analysis.*

Reviewer 3

In this manuscript, Makrooni et al. described a unified framework for gene set analysis. The proposed method considers both the effect size (i.e., log fold change (LFC)) as well as the uncertainty of the effect size estimates from the common DE analysis (i.e., standard error of the LFC). The proposed method used a mixture of Gaussian models containing three components corresponding to up-regulated, down-regulated, and non-DE genes. The authors further utilized the EM algorithm for the parameter estimation and test for differences in these distributions between gene sets. The method is expected to be applicable to a wide range of RNA-seq studies. Generally, the manuscript is written in an organized way. Specific comments are listed below.

Major comments:

1. The authors described two sets of statistical tests for the comparison of the model fitting, which investigate whether the LFC distribution is same or different given the genes in the gene set and those not included. However, there is a lack of detailed description of parameter estimation (i.e., how to estimate μl,πl,$\sigma l2$), And the exact statistical test used (i.E., Likelihood ratio test) in the methods section. The authors should provide the details about the EM algorithm implementation.

   Response*: We thank the reviewer for raising this point. A detailed methodology document has been provided and added to our supplementary materials (S1 Appendix).*

2. The authors performed simulation studies to evaluate the power and accuracy of the proposed method. They first simulated the count data from a negative binomial distribution based on different groups with the parameters estimated from realistic data, then they generated 1000 power simulation settings and 9000 null simulation settings, then combined them to correct for the multiple testing. Finally, the authors compared the power of their proposed method (1DF test) with ORA, GSEA and SAFE. However, there are several concerns about the simulations:

1) In the simulation setting, equal number of DE genes were set to be up- and down-regulated. Is this true in the case of real data? What are the benchmarking results when different number of genes are set to be up- and down-regulated?

Response: Enrichment for upregulated genes and down regulated genes is common but we agree with the reviewer is correct that an equal split is only one of many potential ways that gene sets can be enriched. We have carried out additional simulations for the revised manuscript, with enriched gene sets with different proportions of upregulated and downregulated genes. The results of these simulations are provided in Supplementary Figure 7 of the revised manuscript.

2) In the null simulation setting, what are the performances of different methods in terms of type I error control? i.e., QQ plot / histogram of the p-value from the null simulation setting.

Response: S4 Fig shows the results of the null simulations. The p-value distributions of the null simulations with larger LFC values simulated (as in Fig1) are shown here.

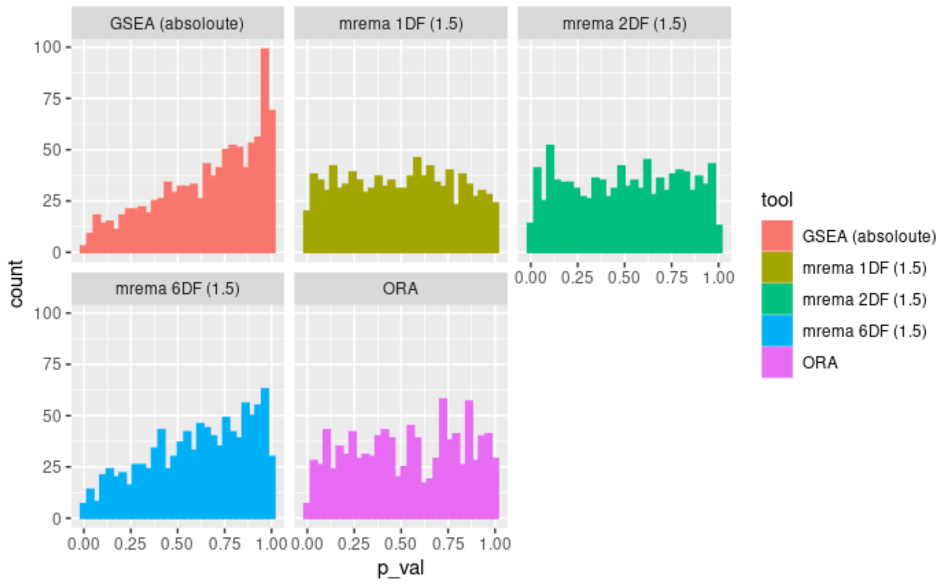

3) The current simulation set the number of genes enriched in each gene set to be 100 among 10000 genes. However, in the real data applications, there are several identified gene sets with gene set size < 100 (i.e., S1 Table). It is important to evaluate the impact of gene set size on the performance of different methods.

Response: The reviewer raises an important point and we have carried out additional simulations to evaluate the impact of gene set size on the performance of the different methods. The results are shown in Supplementary Figure 8 of the revised manuscript.

4) The simulation for evaluating the power and accuracy of 6DF test is lacking. It will be interesting to add a simulation setting on this to investigate the performance of 6DF on the differences in the mixture distribution between the inset and outset.

   Response: The results for the 6DF test have been added for all simulations, in Fig.1, 2, S1-3 Fig and S6-8 Fig.

5) What is the power of the 1DF test at different thresholds for 20 samples with high/low LFC values?

   Response: Supplementary figure 1, 2 and 3 of the revised manuscript now demonstrate the effects of the different thresholds across all simulations.

3. In the TCGA data applications, the authors only displayed the results of 1DF test in supplementary tables, what are the results of 6DF test?

   Response: The results of the 6DF test are now evaluated in the real data analysis in fig.4 and fig. 5. It should be noted that the approach to real data analysis has been revised substantially at the suggestion of other reviewers.

4. In Figure 3, the behavior of ORA on the varying sample size seems to be contradicted with that in the simulation setting in Figure 1C&D. How to interpret this result?

   Response: In Fig.3 the power is based on the adjusted p-values. This meant that only one gene set was available for analysis. This adjusted p-value was very weakly significant for ORA with the full dataset. As such the comparison with the results produced by GSEA, the 1DF and 6DF test was flawed. We thank the reviewer for highlighting this and have revised our approach to the real data analysis (results are shown in Figures 4 and 5 of the revised manuscript).

5. In Figure 3, the performance of 1DF test and 6DF test are very similar, only showing difference when the sample size is < 5. I would suggest the authors to investigate the situation where the 1DF test showed distinct performance from 6DF test, thus guiding users to choose the appropriate test.

   Response: As mentioned above this section is no longer a part of the manuscript but the reviewer is correct to recommend that guidance should be given on the choice of test. This is now given in

the discussion based on the results of the real data analysis and the simulation results, on lines 308-316.

6. One of the limitations of this method is that it cannot deal with the overlaps between the gene sets. The authors should discuss the potential limitation of the proposed method when testing the gene sets that are non-independent.

   Response: We thank the reviewer for this suggestion and have added this limitation to the discussion on lines 279-285, along with potential future directions for accounting for this overlap.

Minor points:

1. I failed to run the tutorial provided on the Github website: https://github.com/osedo/GSA-MREMA. There is no example data (i.e., postdata_overlap) on the Github.

   Response: Example data has been added to the github repository. We have also provided code to allow the download of the real datasets using the GSEABenchmarker package.

2. Line 129 typo "S2 Table and S2 Table".

   Response: Corrected, with many thanks to the reviewer for highlighting this mistake.

3. Figure 3, y axis tittle "Proportion of time xxx".

   Response: We thank the reviewer for pointing out this error. This figure is no longer part of manuscript.
