# Supplementary material for: Random-effects meta-analysis of effect sizes as a unified framework for gene set analysis
Source: PLoS Comput Biol. 2022 Oct 5;18(10):e1010278. doi: 10.1371/journal.pcbi.1010278 (PMC9576052; doi:10.1371/journal.pcbi.1010278)
Supplement: Attachment — Submitted filename: Response_to_reviewers.pdf [file pcbi.1010278.s019.pdf]

Changes to manuscript

1. Missing citation included.
2. Figures nor ordered correctly.
3. References all withing parentheses.
4. Overlong sentences partitioned for clarity.
5. Changed notation for gene set membership in the methods to match that of the appendix.
6. Added details regarding the null simulations in the methods.
7. Changed colours in the figures to avoid confusion.
8. Reordered panels in Fig. S4.
9. Changed labels in Fig.3 and Fig.S5-7 to improve clarity.
10. Updated GitHub; added information on input, parameters, and how to interpret output.

Reviewer #1

The authors addressed my comments and suggestions. I only have a few more minor comments as follows.

1. Line 17 the literature number for ORA is missing.
   - *Response: This has been included with thanks for highlighting the error.*

2. In Fig.1B and Fig.1C, it's hard to distinguish ORA (threshold) and MERMA-6DF and the colors are too similar.
   - *Response: The colour of MREMA-6DF has been changed in all figures in which it appears.*

3. Fig.3 is mentioned before Fig.2 (line 112)
   - *Response: We thank the reviewer for highlighting this error. Fig2 is now referenced ahead of Fig.3 on line 104*

4. Fig.1 and Fig.2 large/small efect sizes, does it mean large/small log fold change values?
   - *Response: The reviewer is correct; effect sizes are log fold change value in the context of differential expression.*

5. Fig.S1 shows that if we use a small threshold for LFC when the LFC are large, ORA actually has the best power. The authors should provide some explanation about this. Is it because ORA has higher FPR (Fig. S4 cd) when the null simulations contains high LFC? Also, I would suggest the authors to switch the order of Fig.S4 (ab) with Fig.S4(cd) to be consistent with the order of Fig.S1/Fig.S2 and Fig.1/Fig.2, where the results show in order of high/low LFC respectively.
   - *Response: The reviewer is correct that with a fold-change threshold of 1.1 ORA and the simulation parameters in Fig. S1 (A,B,C) ORA appears to have more power than the*

methods we propose. In fact, this illustrates an essential difference between our method and ORA. For these simulations the proportions of the two distributions beyond the thresholds shown in Fig S1A are quite similar, but the proportion of the distributions in the tails (i.e. further from the origin) are different. As sample numbers grow, genes that are less far from the origin become statistically significant in the DE analysis and ORA becomes less influenced by the tails of the distribution. This results in the undesirable property of the power of ORA appearing to decrease with increasing sample size. For example, for the parameter values corresponding to 129% enrichment in DE genes in the inset, ORA has ~50% power for 20 samples and ~58% power for 40 samples. When these simulations were run with 400 samples, the power of ORA was ~38%. We have reordered the panels in Fig.S4 as suggested in order to be consistent with other figures as suggested.

6. I would suggest using the name in Fig.S5 as MERMA-1DF instead of GMM to be consistent with other plots.
    - *Response: The names in Fig.S5 have been altered in order to be consistent with other plots. Fig.3 in the main manuscript has also been updated.*

Reviewer #2

This revised version gives me much more confidence. I only have a few very minor suggestions:

1. Authors need to put more effort into polishing manuscript writing. For example, Fig. 2 is cited after Fig. 3 . A LOT of figure citations do not appear within the parenthesis (e.g., lines 105, 116, 154 etc.). There are still a few long, complicated and fragmental sentences. Those are very hard to follow (e.g., lines 196-199).
    - *Response: We thank the reviewer for highlighting these errors. Fig. 2 is now referenced on line 104 before Fig. 3. All citations have been placed within parentheses. The sentence on lines 196-199 has been re-worded.*

2. Methods comparison figures are not consistent. Some figures contain more or fewer methods (e.g., Supplementary Figures 1-3 do not have GSEA. Supplementary Figure 7 adds GSEA and GSEA (absolute)). Authors should make the methods to be compared and the color settings consistent for easy comparison.
    - *Response: We thank the reviewer for highlighting inclusion of an additional method to Supplementary Figure 7, and the confusion created by our choice of label. We have renamed GSEA to GSEA (directional). We have only included this tool in Supplementary figures 6 and 7 as these are the only simulation scenarios where DE genes are not evenly split between upregulated and downregulated. The comparison of a directional tool in Fig.1-2 and Supplementary figures 1-3, and 8 would disadvantage this tool given the even split between upregulated and downregulated genes. This could in turn create an*

impression that the directional GSEA functions poorly while in reality it is testing for a different kind of enrichment.

3. GitHub software also needs further polishing to include the parameter explanation, input data format introduction, and output data interpretation/explanation.
    - *Response: The GitHub page has been updated.*

Reviewer #3

The authors have addressed the majority of my concerns by adding new evaluation results and more detailed descriptions of the methods. I just have some minor comments:

1. In the Materials and methods part of the main manuscript, the authors used qi as the binary value representing the gene set membership. But in the S1 Appendix -Methodology, the zi is used to indicate the gene set membership. Could the authors make them consistent?
    - *Response: We thank the reviewer for highlighting this inconsistency and we have amended the main manuscript so that z_i represents gene set membership.*

2. In the alternative model for the 1DF test (S1 Appendix Eq. 29), clearer and consistent notations/definitions are needed for the $\pi 1$ and $\pi 2$. Do they represent the proportion of non-DE inside and outside of the gene set respectively? However, in the null model defined in S1 Appendix Eq. 2, $\pi 1$, $\pi 2$, and $\pi 3$ correspond to the proportion of genes to be upregulated, downregulated, and not affected by the group. Also, in the S1 Appendix Eq. 3, $\pi 2$ represents the proportion of non-DE genes as $\mu 12$=$\mu 22$=0 and $\sigma 12$,$\sigma 22$ are set to be fixed. These three sets of notations might confuse the readers due to their inconsistency. Could the authors elaborate more on the $\pi 1$, $\pi 2$, and $\pi 3$ in each alternative model and make them consistent (1DF, 2DF, and 6DF tests)?
    - *Response: We thank the reviewer for their suggest and we have amended S1 appendix to clarify what is meant by π in each approach.*

3. Description of the null simulations are missing in the Simulations section on pages 12-13
    - *Response: We have added details regarding the null simulations in lines 406-408.*

4. There is a typo on the Github tutorial webpage: lens <- lengths(kegg.gs.overalp). “kegg.gs.overalp” should be “kegg.gs.overlap”.
    - *Response: This has been corrected with thanks to the reviewer for highlighting this error.*

5. Line 112 on page 4, Figure 3 is mentioned before Figure 2.

- *Response: We thank the reviewer for highlighting this error. Fig2 is now referenced ahead of Fig.3 on line 104*
