# Supplementary material for: Random-effects meta-analysis of effect sizes as a unified framework for gene set analysis
Source: PLoS Comput Biol. 2022 Oct 5;18(10):e1010278. doi: 10.1371/journal.pcbi.1010278 (PMC9576052; doi:10.1371/journal.pcbi.1010278)
Supplement: Attachment — Submitted filename: renamed_9e3f0.docx [file pcbi.1010278.s014.docx]

**Review of “Random-effects meta-analysis of effect sizes as a unified framework for gene set analysis”**

The authors propose a novel framework for testing whether there is a difference in the distribution of differentially expressed (DE) effect sizes for the gene set of interest against the background gene set. Unlike the existing gene set analysis (GSA) methods, the proposed framework is sensitive to a wider range of signals, such as different proportion of the DE gene (1DF test), or that the proportion of DE gene is the same but the size of DE effect is different (6DF test). Specifically, the authors utilize the Gaussian mixture model (GMM) to model the effect sizes for non-DE genes and positive and negative DE genes in a given gene set. Following the random-effect meta-analysis framework, they incorporate the uncertainty of effect size estimates from each gene to increase the power of the proposed test. The Expectation-maximization algorithm is implemented to estimate the parameters in the GMM. The authors show that the proposed method is more powerful and robust than GSEA, ORA and SAFE approaches via simulation studies and real data analysis.

Overall, the manuscript is interesting and shows the improvements in power by combining the random-effect meta-analysis framework into GSA. I have several major comments/questions about the manuscript for the authors:

1. It would be easier for readers to understand/follow the proposed framework if the authors provide more details about the methodology in the Methods section or in the supplementary materials. The following are some examples that can be elaborated on for clarity:
(a) In GMM subsection, it is unclear of what the weight $\pi_{\mathcal{l}}$ means here. Can the weight $\pi_{\mathcal{l}}\mathcal{, l=}1, 2, 3$ be viewed as the proportional of non-DE, up-regulated, and down-regulated DE genes in the gene set? Or is this weight $\pi_{\mathcal{l}}$ equivalent to weight adjusting for uncertainty in each gene (Eq. 5), $W_{i}=\frac{1}{\sigma_{i}^{2}+\sigma_{\mathcal{l}}^{2}}$.
(b) Does the model parameters set include $\theta_{0}=\left( \pi_{\mathcal{l}},\mu_{\mathcal{l}}, \sigma_{\mathcal{l}} \right)\mathcal{, l=}1, 2, 3$ (under the null hypothesis) and $\theta=\left( \pi_{1\mathcal{l}},\mu_{1\mathcal{l}}, \sigma_{1\mathcal{l}}, \pi_{2\mathcal{l}},\mu_{2\mathcal{l}}, \sigma_{2\mathcal{l}} \right)\mathcal{,l=}1, 2, 3$ (under the alternative hypothesis) that need to be estimated? Is $\sigma_{i}^{2}$ the input data and viewed as fixed value? Assume there are $m_{1}$ genes in the inset and $m_{2}$ genes in the outset, does the algorithm only use the effect size estimates data (or LFC) from these $m_{1}$ genes to estimate $(\pi_{1\mathcal{l}},\mu_{1\mathcal{l}}, \sigma_{1\mathcal{l}})$?
(c) In lines 327-329, it says “the weights assigned to the non-DE genes …”. Is the same estimation procedure conducted for the positive/negative DE in the inset/outset?
(d) The implementation of EM algorithm is not clear. It would be very helpful if the authors provide details about their use of the EM algorithm or some pseudo-code to help readers better understand and use their framework.
(e) It is unclear how to place the constraint on the parameters of GMM (lines 333-335). The reasons on how to set this constraint are not elaborated and the choice of values of 0.25/0.95 seems arbitrary.
(f) It would be helpful if the authors can give more details about hypothesis testing. The authors use EM algorithm to estimate the parameters in GMM after incorporating the with-gene uncertainty. However, it is unclear what statistical test is conducted in the 1DF or 6DF test, and what the corresponding test statistics are. From lines 64-66, it seems that the likelihood ratio test is used, but it is not stated explicitly. Moreover, in line 158, the authors only report a p-value of “P=$9.2\times{10}^{-60}$” but give no mention of what statistical test was used.
(g) Similarly, for the 95% confidence interval on the proportion of DE genes (line 74-75), how was it constructed, especially when then sample size is quite small (n=5~40)?
2. One of the more major innovations of the proposed framework is that it can identify differences in the mixture distribution between the inset and outset. However, this claim is not supported by any simulation studies or real data set. The performance (such as power and false positive rate) of the proposed 6DF test is unknown. Additional simulation studies should be considered and presented, where the only differences are in the signs or the sizes of the DE effects while the proportion of DE genes in the inset/outset are the same. Such a simulation study provides evidence on how the 6DF test identifies gene sets with differences in distribution in DE effects and what gain exists in using the proposed framework over the 1DF test and other existing methods.
3. The proposed method requires a pre-specified threshold $\tau$ to define DE (line 333). The choice of $\tau$ seems to impact the power for the proposed method (Fig 2) and ORA (S4 Fig) largely when there are different standard deviations between inset and outset. It would be helpful if authors provide more guideline of how to choose this value in practice since the true distribution of inset/outset is unknown. Would it further increase power if the proposed method used a grid search to find the optimal threshold $\tau$, a common practice implemented in SKAT-O (Lee et al 2012)?

*Lee, S., Emond, M.J., Bamshad, M.J., Barnes, K.C., Rieder, M.J., Nickerson, D.A., Team, E.L.P., Christiani, D.C., Wurfel, M.M., Lin, X. and NHLBI GO Exome Sequencing Project, 2012. Optimal unified approach for rare-variant association testing with application to small-sample case-control whole-exome sequencing studies. The American Journal of Human Genetics, 91(2), pp.224-237*.

Other minor comments/questions about the manuscript are listed below:

1. In the introduction, since the authors mention some ranking-based approaches may be computationally expensive, the paper would benefit from the authors sharing comparison results on the computational time of the proposed method with existing approaches.
2. Some sentences are too long and wordy putting an unnecessary burden on readers. For example, the sentence in lines 359-363 may be easier to follow if it was split into two or more clear and concise sentences.
3. The legend of supplementary tables should be more specific. For example, in S1 Table “Gene sets identified by 1DT test with a threshold of 1.5”. It’s not clear to know what the threshold is about without referring to the main text.
4. The order of figures is not in reading order, e.g., the main text mentions S4 Fig before S2/S3 Fig. Also, Fig 1B is not mentioned by the main text. It’s not clear how the proportion DE is calculated in the Fig 1B table. Is it the average empirical proportion of DE genes in the simulation studies or is it the area under the curve for the tail of the normal distribution?
5. Legend is inconsistent and mislabeled. For example, the proposed method is named as 1DFT1.5 in the Fig 1, while in S2 Fig and S3 Fig, it’s named as T1.5-DF7.
6. How many genes were generated in the RNA-Seq gene expression data? What’s the size of each non-overlapping gene sets? Also, how was the RNA-Seq data used in the simulation (since later LFC was generated from normal distribution)?
7. It is unclear what the LFC power simulation settings are (lines 378-380). In this setting, each power simulation contains one truly enriched gene set (with 100 genes) and the outset contains 9900 genes and is repeated 1000 times. So, will the power simulation return 1000 p-values in total? Where does the 9000 non-enriched sets p-values come from? It will also help readers to understand if authors would specify explicitly how they calculate the power and false positive rate for each approach in the simulation studies.
8. In TCGA data analysis, was group membership included as an interaction term when estimating the fold change between different patients (lines 398-399)?
9. For the R code on Github, the example data set is not available and so the example code cannot be run.
10. PLOS Computational Biology uses ICMJE reference style, and the authors should use the abbreviation instead of full name of the journal, e.g., Genet Mol Res instead of Genetics and Molecular Research.
