# Supplementary material for: Random-effects meta-analysis of effect sizes as a unified framework for gene set analysis
Source: PLoS Comput Biol. 2022 Oct 5;18(10):e1010278. doi: 10.1371/journal.pcbi.1010278 (PMC9576052; doi:10.1371/journal.pcbi.1010278)
Supplement: Attachment — Submitted filename: PCOMPBIOL-D-22-00849_Review_0624.docx [file pcbi.1010278.s016.docx]

In this manuscript, Makrooni et al. described a unified framework for gene set analysis. The proposed method considers both the effect size (i.e., log fold change (LFC)) as well as the uncertainty of the effect size estimates from the common DE analysis (i.e., standard error of the LFC). The proposed method used a mixture of Gaussian models containing three components corresponding to up-regulated, down-regulated, and non-DE genes. The authors further utilized the EM algorithm for the parameter estimation and test for differences in these distributions between gene sets. The method is expected to be applicable to a wide range of RNA-seq studies. Generally, the manuscript is written in an organized way. Specific comments are listed below.

**Major comments:**

1. The authors described two sets of statistical tests for the comparison of the model fitting, which investigate whether the LFC distribution is same or different given the genes in the gene set and those not included. However, there is a lack of detailed description of parameter estimation (i.e., how to estimate $\mu_{l}, \pi_{l}, \sigma_{l}^{2}$), and the exact statistical test used (i.e., likelihood ratio test) in the methods section. The authors should provide the details about the EM algorithm implementation.
2. The authors performed simulation studies to evaluate the power and accuracy of the proposed method. They first simulated the count data from a negative binomial distribution based on different groups with the parameters estimated from realistic data, then they generated 1000 power simulation settings and 9000 null simulation settings, then combined them to correct for the multiple testing. Finally, the authors compared the power of their proposed method (1DF test) with ORA, GSEA and SAFE. However, there are several concerns about the simulations:
1) In the simulation setting, equal number of DE genes were set to be up- and down- regulated. Is this true in the case of real data? What are the benchmarking results when different number of genes are set to be up- and down-regulated?
2) In the null simulation setting, what are the performances of different methods in terms of type I error control? i.e., QQ plot / histogram of the *p-*value from the null simulation setting.
3) The current simulation set the number of genes enriched in each gene set to be 100 among 10000 genes. However, in the real data applications, there are several identified gene sets with gene set size < 100 (i.e., S1 Table). It is important to evaluate the impact of gene set size on the performance of different methods.
4) The simulation for evaluating the power and accuracy of 6DF test is lacking. It will be interesting to add a simulation setting on this to investigate the performance of 6DF on the differences in the mixture distribution between the inset and outset.
5) What is the power of the 1DF test at different thresholds for 20 samples with high/low LFC values?
3. In the TCGA data applications, the authors only displayed the results of 1DF test in supplementary tables, what are the results of 6DF test?
4. In Figure 3, the behavior of ORA on the varying sample size seems to be contradicted with that in the simulation setting in Figure 1C&D. How to interpret this result?
5. In Figure 3, the performance of 1DF test and 6DF test are very similar, only showing difference when the sample size is < 5. I would suggest the authors to investigate the situation where the 1DF test showed distinct performance from 6DF test, thus guiding users to choose the appropriate test.
6. One of the limitations of this method is that it cannot deal with the overlaps between the gene sets. The authors should discuss the potential limitation of the proposed method when testing the gene sets that are non-independent.

**Minor points:**

1. I failed to run the tutorial provided on the Github website: <https://github.com/osedo/GSA-MREMA>. There is no example data (i.e., postdata_overlap) on the Github.
2. Line 129 typo “S2 Table and S2 Table”.
3. Figure 3, y axis tittle “Proportion of time xxx”.
