# Supplementary material for: Random-effects meta-analysis of effect sizes as a unified framework for gene set analysis
Source: PLoS Comput Biol. 2022 Oct 5;18(10):e1010278. doi: 10.1371/journal.pcbi.1010278 (PMC9576052; doi:10.1371/journal.pcbi.1010278)
Supplement: Attachment — Submitted filename: PCOMPBIOL-D-22-00849_R2_Review.docx [file pcbi.1010278.s018.docx]

The authors have addressed the majority of my concerns by adding new evaluation results and more detailed descriptions of the methods. I just have some minor comments:

1. In the Materials and methods part of the main manuscript, the authors used $q_{i}$ as the binary value representing the gene set membership. But in the S1 Appendix -Methodology, the $z_{i}$ is used to indicate the gene set membership. Could the authors make them consistent?
2. In the alternative model for the 1DF test (S1 Appendix Eq. 29), clearer and consistent notations/definitions are needed for the $\pi_{1}$ and $\pi_{2}$. Do they represent the proportion of non-DE inside and outside of the gene set respectively? However, in the null model defined in S1 Appendix Eq. 2, $\pi_{1}$, $\pi_{2}$, and $\pi_{3}$ correspond to the proportion of genes to be upregulated, downregulated, and not affected by the group. Also, in the S1 Appendix Eq. 3, $\pi_{2}$ represents the proportion of non-DE genes as $\mu_{12}=\mu_{22}=0$ and $\sigma_{12}, \sigma_{22}$ are set to be fixed. These three sets of notations might confuse the readers due to their inconsistency. Could the authors elaborate more on the $\pi_{1}$, $\pi_{2}$, and $\pi_{3}$ in each alternative model and make them consistent (1DF, 2DF, and 6DF tests)?
3. Description of the null simulations are missing in the Simulations section on pages 12-13
4. There is a typo on the Github tutorial webpage: lens <- lengths(kegg.gs.overalp). “kegg.gs.overalp” should be “kegg.gs.overlap”.
5. Line 112 on page 4, Figure 3 is mentioned before Figure 2.
